# ^16^O poor cosmic spherules from near-Earth CY chondrite asteroids

**DOI:** 10.1126/sciadv.aed6340

**Published:** 2026-06-26

**Authors:** Matthias Van Ginneken, Steven Goderis, Matthew J. Genge, Guido Jonker, Ryoga Maeda, Penelope J. Wozniakiewicz, Luigi Folco, Martin D. Suttle, Ian A. Franchi, Xuchao Zhao, Akira Yamaguchi, Sophie Decrée

**Affiliations:** ^1^ School of Engineering, Mathematics and Physics, University of Kent, Canterbury CT2 7NZ, UK.; ^2^ Archaeology, Environmental Changes, and Geo-Chemistry, Vrije Universiteit Brussel, 1050 Brussels, Belgium.; ^3^ Department of Earth Science and Engineering, Imperial College London, London SW7 2AZ, UK.; ^4^ Department of Earth Sciences, Faculty of Science, Vrije Universiteit Amsterdam, 1081 HV Amsterdam, Netherlands.; ^5^ Submarine Resources Research Center (SRRC), Japan Agency for Marine-Earth Science and Technology (JAMSTEC), Yokosuka 237-0061, Japan.; ^6^ Dipartimento di Scienze della Terra, Università di Pisa, 56126 Pisa, Italy.; ^7^ School of Physical Sciences, The Open University, Milton Keynes MK7 6AA, UK.; ^8^ National Institute of Polar Research, Tokyo 190-8518, Japan.; ^9^ Belgian Geological Survey, Royal Belgian Institute of Natural Sciences, 1000 Brussels, Belgium.

## Abstract

Approximately 10% of cosmic spherules—microscopic extraterrestrial particles that melt upon atmospheric entry and dominate the influx of astromaterials to Earth—exhibit anomalous oxygen isotopic compositions, suggesting an asteroidal source not represented in current meteorite collections. We introduce a a previously unidentified subset of micrometeorites, the sulfur-rich cumulate olivine (SCumPo) cosmic spherules, characterized by cumulate textures evidencing the settling of olivine crystals, and oxygen-16 (^16^O)–poor bulk signatures. The systematically nickel-poor olivine phenocrysts, frequent iron-nickel-sulfur droplets, unusually sulfur-rich mesostasis, and a virtual absence of magnetite all point to unusually highly reducing conditions during atmospheric entry, which may reflect unusual precursor mineralogy. Numerical modeling of olivine settling under deceleration speeds of ~14 to 17 kilometers per second suggests high-eccentricity precursor orbits (*e* > 0.2), incompatible with typical main-belt asteroid sources. These findings point to a previously unsampled, primitive, sulfide-rich CY-like near-Earth asteroid, which represents a “missing” meteorite parent body that contributes distinctive ^16^O-poor cosmic dust to Earth.

## Teaser

Unique cosmic dust reveals a link to primitive near-Earth asteroids missing from meteorite collections.

## INTRODUCTION

Micrometeorites are dust-sized particles (~10 μm to 2 mm in size) that survive atmospheric entry and dominate the flux of extraterrestrial matter accreting to Earth in terms of mass (*1*, *2*). Petrological geochemical and isotopic studies have shown that the vast majority of micrometeorites >100 μm likely come from asteroids (*3*–*8*). The modern flux of micrometeorites to Earth’s surface is commonly interpreted as resulting from recent collisional processes; the Poynting-Robertson effect then efficiently drives dust particles sunward, making micrometeorites effectively microsamples from asteroids in the Main Asteroid Belt between Mars and Jupiter [e.g., (*9*)]. A major interest in the study of micrometeorites is identifying the nature of their parent bodies, in particular those that might not be related to known meteorites.

Cosmic spherules account for 70 to 90% of all micrometeorites and represent the group that suffered the highest degree of thermal alteration during atmospheric entry, resulting in the near-complete melting of the precursor dust particles (*10*). Consequently, primary features such as mineralogy and chemistry are strongly altered or obliterated, except for rare relict phases sufficiently refractory to resist peak temperatures in excess of ~1000°C [typically forsteritic olivine (*11*, *12*)]. As such, identifying the precursors of cosmic spherules is challenging, and a direct comparison with larger objects like meteorites is largely inconclusive. On the basis of bulk oxygen isotope studies, over 90% of silicate-dominated (S-type) cosmic spherules larger than 200 μm are linked to chondritic sources derived from asteroidal material (*5*, *13*–*16*). Although the majority can be associated with primitive carbonaceous, more evolved ordinary chondrites and, to a much lesser extent, to differentiated achondritic material, a distinct minority exhibit ^16^O-poor isotopic signatures that are inconsistent with known meteorite groups. To date, 35 such cosmic spherules have been identified of the 399 S-type spherules analyzed (*8*, *11*, *17*–*19*). This peculiar isotopic group [Δ^17^O ≈ +2 per mil (‰); δ^18^O ≈ 20 to 40‰], labeled “Group 4” (*5*), has long been thought to sample a parent body that is not represented in meteorite collections. Recent studies have shown that rare, large unmelted micrometeorites, essentially fine-grained phyllosilicate-rich particles, exhibit ^16^O-poor values, suggesting that these may be the unmelted counterparts of cosmic spherules in isotopic Group 4, i.e., the ^16^O-poor cosmic spherules (*20*, *21*).

Although oxygen isotopes and, to a limited extent, bulk geochemistry can help identify potential parent bodies for cosmic spherules, these criteria do not provide information on the dynamical history of cosmic spherules, in particular their orbital parameters, which could prove critical to identifying the source regions of micrometeorites. In one particular case, however, the mineralogical and textural properties of a subset of cosmic spherules do provide information on the orbital parameters of their precursors, including eccentricity and encounter velocity (*12*). This subset is termed CumPo after their characteristic cumulate olivine porphyritic texture, characterized by clustered olivine phenocrysts that increase in size from one side of the spherule to the other. Genge *et al.* (*12*) suggested that this porphyritic texture results from the survival of relict forsterite (Mg-rich olivine), which acts as a crystallization site for olivine phenocrysts that then grow freely in the silicate melt. The rapid settling of olivine crystals—resulting in the cumulate texture—suggests unusually strong deceleration in the atmosphere and, as a result, entry velocities of ~16 km s^−1^ for the incoming particle. This velocity corresponds to orbital eccentricities in excess of ~0.3, which is higher than those of most asteroidal dust bands commonly associated with cosmic dust. It suggests that a subset of cosmic spherules exhibits unusual orbital parameters that may point to an atypical parent body.

Here, we report that most ^16^O-poor cosmic spherules are also CumPo cosmic spherules, and together they represent an unusual subset of cosmic dust. Crucially, the distinct characteristics of these spherules indicate they are derived from a material that is currently not represented in our meteorite collections. We present the petrological, mineralogical, and isotopic properties of these particles, as well as numerical simulations of olivine settling during atmospheric entry, to demonstrate that they may originate from a single parent body consistent with a near-Earth asteroid.

## RESULTS

For this study, we examined 10 CumPo cosmic spherules: five from the Sør Rondane Mountains (SRM) Antarctic micrometeorite collection (prefix WN for Walnumfjellet Nunatak), one from the Larkman Nunatak (LK) collection, and four from the newly described Budel urban (BU, prefix GMM) micrometeorite collection (*15*, *22*, *23*). Scanning electron microscopy (SEM) images of the polished sections of all particles studied are shown in fig. S1. The CumPo texture is clearly evident in most cosmic spherules (fig. S1, A to F and I), whereas it is less obvious in three of the BU particles (fig. S1, G, H, and J). In the latter spherules, the spatial distribution of olivine crystals is nonetheless consistent with a cumulate texture, with increasing abundance of crystals toward one side, suggesting that the weaker appearance is an artifact of the sectioning orientation. All 10 spherules share key petrological and mineralogical parameters that justify their inclusion together in this study.

**Fig. 1.**
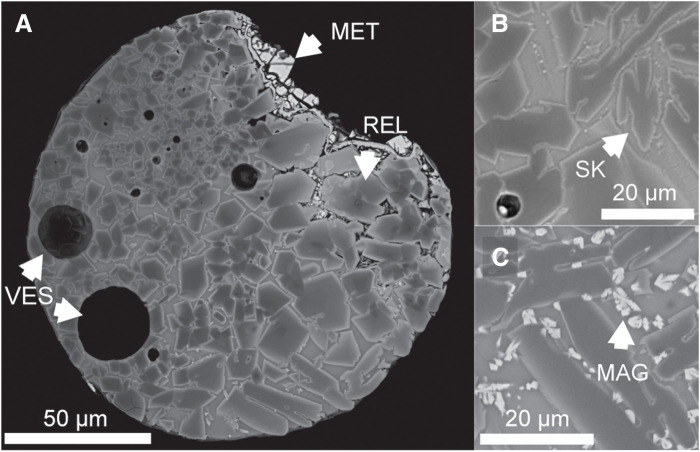
BSE SEM images of SCumPo cosmic spherule WN-790. (**A**) Section across an entire SCumPo particle, showing the typical cumulate olivine texture: a fine-grained μPo region in the top left (with many small olivine crystallites and vesicles) gradually transitions to a coarser Po texture in the bottom right. Relict olivine (REL), Fe-Ni-S metal bead (MET), and vesicles (VES) are labeled. In this example, the metal bead has been partially altered by terrestrial weathering during storage in Antarctic ice (*43*). (**B**) Skeletal olivine (SK) crystal within a SCumPo cosmic spherule. (**C**) Interstitial magnetite (bright phase) in a typical porphyritic olivine (Po) cosmic spherule for comparison. Note that the thin section may not capture the true aerodynamic axis of the spherule. Thus, the apparent offsets of the metal bead and cumulate texture here do not necessarily contradict a deceleration-driven cumulate pattern.

In the studied particles, olivine crystals appear as microphenocrysts and euhedral phenocrysts, and in some cases, skeletal olivine forms are observed (Fig. 1, A and B). CumPo cosmic spherules are distinguished by (i) their unique cumulate (settled crystal) texture, (ii) the frequent occurrence of relict forsteritic olivine grains (marked “REL” in Fig. 1A), (iii) the absence of magnetite in the glassy mesostasis, contrary to normal porphyritic olivine (Po) cosmic spherules (Fig. 1C), and (iv) the presence of vesicles in regions with microporphyritic (μPo) texture (labeled “VES” in Fig. 1A). In addition, small Fe-Ni-S metal beads are observed in about 10% of the CumPo spherules (example indicated as “MET” in Fig. 1A).

For all WN particles (and some others), one side of the spherule cross section shows a μPo texture with abundant vesicles, whereas the opposite side shows a more typical Po texture with few or no vesicles (see fig. S1, A to E). In effect, each CumPo spherule is internally heterogeneous: A vesicle-rich fine-grained region coexists with a relatively vesicle-poor coarser-grained region. This dichotomy is evident, for example, in Fig. 1A (top left versus bottom right). Relict forsterite crystals, when present, tend to be located near the periphery of the spherule (often at the boundary of the coarse-grained region). Such spatial segregation of textures and relict phases is consistent with considerable crystal settling and possibly rotation of the molten droplet during flight, with spin axis orientation likely playing a role in maintaining a stable aerodynamic state during atmospheric entry (*12*). In summary, the CumPo cosmic spherules identified here are characterized by a combination of features—textural, mineralogical, chemical, and isotopic—that are highly unusual compared to the general cosmic spherule population.

Apart from their textural and isotopic properties, the CumPo cosmic spherules studied here also exhibit petrological and geochemical features that set them apart from most previously documented Po and μPo cosmic spherules. A notable observation is the virtual lack of magnetite in the glassy mesostasis of these particles. Magnetite is a common accessory phase in S-type cosmic spherules, including normal Po/μPo types (*10*). Upon melting of ordinary chondritic particles during atmospheric entry, magnetite readily forms by oxidation of Fe^2+^ in the silicate melt, even at the relatively low oxygen fugacity of the upper mesosphere (*24*, *25*). In the present particles, however, magnetite is absent or extremely scarce. Correspondingly, 5 of the 10 spherules contain evidence of immiscible Fe-Ni-S metallic droplets (observable as Fe-Ni-S beads in the polished section; see examples in fig. S1 and analyses in table S2). The remaining five spherules do not show discrete metal beads in the sectioned plane; however, they exhibit a pronounced increase in Fe content on one side of the particle [visible as higher backscattered electron (BSE) brightness), which suggests that a metallic bead was present but not intersected by the plane of the section.

Neoformed olivine phenocrysts—olivine crystals that crystallize in the silicate melt—are consistently Ni-poor: Their NiO contents are below ~0.5 wt % (fig. S3 and table S3). Relict olivine grains that survive melting during atmospheric entry, when present, exhibit a wide range of Fe content (Fo_82–98_) and systematically Ni-poor. For comparison, typical porphyritic cosmic spherules can contain either Ni-poor or Ni-rich olivine populations depending on precursor metal abundance and oxidation conditions (*11*). The uniformly low Ni content of both relicts and neoformed olivines in our particles is unusual. We also measured the major- and minor-element composition of the interstitial glass in each spherule (tables S4 and S5). Unexpectedly, all CumPo cosmic spherules show measurable concentrations of sulfur in their glass, whereas sulfur is typically below detection in cosmic spherules due to nearly complete volatilization during entry heating (*26*). In our CumPo samples, S is detected at levels of a few weight % (on the order of 1 to 3 wt % SO_3_ equivalent in the glass by electron probe analyses), which is extraordinarily high for cosmic spherules. Given their distinctive combination of a cumulate texture, Ni-poor olivine, the presence of Fe-Ni-S metal droplets, a lack of magnetite, and S-rich glass, we designate this subset of cosmic spherules as sulfur-rich cumulate olivine cosmic spherules, abbreviated SCumPo cosmic spherules.

**Fig. 2.**
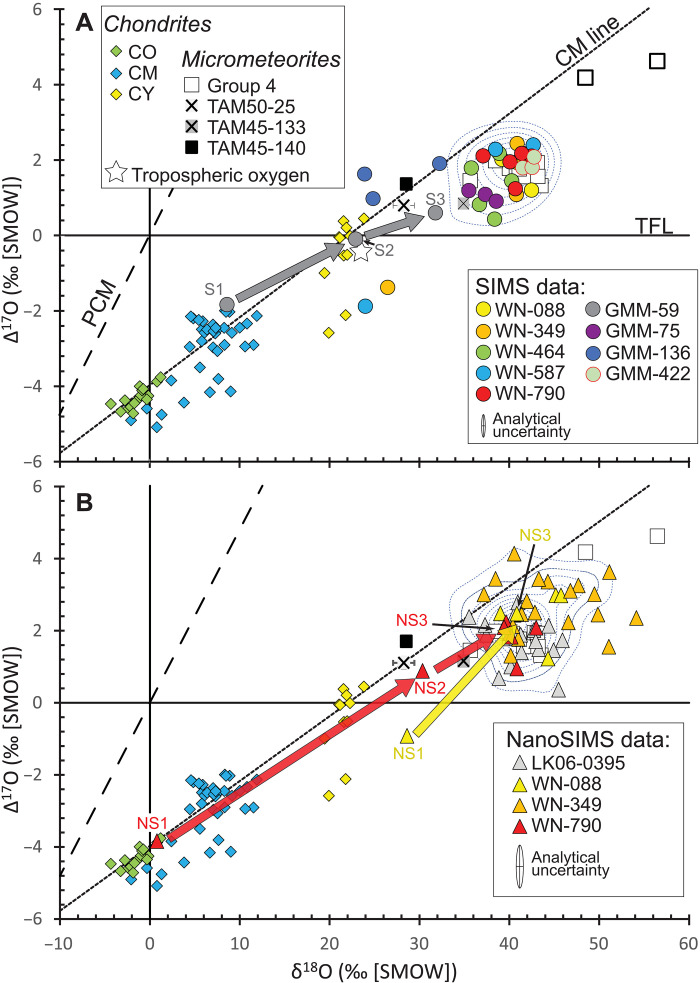
Oxygen three-isotope plot of SCumPo cosmic spherules. (**A**) SIMS (circles) and (**B**) NanoSIMS (triangles) analyses. Each colored symbol marks an individual analysis spot, with color coding used to distinguish between different cosmic spherules; arrows indicate the shifts in isotopic composition from relict olivine cores to their adjacent neoformed olivine overgrowths in specific particles [GMM59 in (A); WN-088 and WN-790 in (B)]. Labels next to data points correspond to analysis numbers in table S1. For reference, data for established chondrite groups are shown as colored diamonds, and the composition of terrestrial stratospheric O_2_ is marked by the black cross (*44*). The following reference lines are plotted: the TFL, the primary chondrule mineral (PCM), and the CM mixing line (*45*). Chondrite data are from (*20*, *21*, *30*, *45*–*60*). SMOW, standard mean ocean water.

The oxygen isotopic compositions of the SCumPo spherules were determined by secondary ion mass spectrometry (SIMS) and NanoSIMS (see Materials and Methods). Six particles (five WN and one LK) were analyzed by SIMS; four GMM particles were analyzed using the same analytical protocol and first reported by Jonker *et al.* (*19*). The three-isotope oxygen results are listed in table S1 and presented in Fig. 2. SIMS point analyses reflect either single olivine crystals (where grains exceeded the ~15-μm spot size) or a mixture of olivine and interstitial glass, as indicated in table S1. Individual neoformed and relict olivine crystals—typically smaller than 10 μm—were analyzed in two WN samples using NanoSIMS.

Most SIMS data points (Fig. 2A) cluster narrowly in the isotopic Group 4 region of the oxygen three-isotope diagram for cosmic spherules that are ^16^O-poor relative to any known meteorite group (*5*). Similarly, the NanoSIMS data for SCumPo spherules (Fig. 2B) show predominantly ^16^O-poor values, except that particle WN-088 has one analysis consistent with the CY chondrite field (Δ^17^O ~ 0‰) and WN-790 has one point plotting near CO/CM chondrite values (Δ^17^O ~ −2‰). Within individual SCumPo spherules, the spread of isotope data can be substantial: Variations up to several per mil in δ^18^O and Δ^17^O are observed between different spots (e.g., between relict olivine and surrounding glass in the same particle). Nonetheless, most measured points lie in the general ^16^O-poor range above the terrestrial fractionation line (TFL; for context, typical carbonaceous chondrites have Δ^17^O ≈ −2‰ to 0‰, and ordinary chondrites ≈ +1‰). The presence of a few analyses with negative Δ^17^O in these spherules is highly unusual for cosmic spherules and will be discussed further below.

## DISCUSSION

### The SCumPo cosmic spherules

The notably reduced mineralogy and chemistry of the SCumPo cosmic spherules provide important insights into their formation conditions and precursor material. The (near total) absence of magnetite in these spherules necessitates that extremely reducing conditions prevailed during their atmospheric entry heating. In typical S-type micrometeorites, magnetite forms readily as molten silicate droplets equilibrate with even trace amounts of atmospheric oxygen (Fig. 1C) (*24*). In the SCumPo spherules, by contrast, iron remains in reduced form (metallic or ferrous Fe^2+^ in silicate) rather than oxidizing to ferric iron (Fe^3+^) to form magnetite (Fe_3_O_4_). One mechanism to achieve such low oxygen fugacity is the presence of abundant carbon in the precursor: As the particle melts, carbon can pyrolyze and react with atmospheric O_2_, forming CO/CO_2_ and thereby consuming oxygen before it can oxidize Fe (*27*). Another potent reducing agent is sulfur, which can vaporize as SO*_x_* gas (primarily SO_2_) during entry, likewise scavenging oxygen (*25*, *28*). The SCumPo spherules show clear evidence for both these reducing agents: They contain Fe-Ni-S metallic droplets (indicating a metal- and sulfide-bearing precursor, likely rich in C and/or S) and retain exceptionally high S in their silicate glass (table S4), consistent with an oxygen-poor melt environment. The presence of a few weight percent of S in the droplets’ mesostasis is highly unusual in cosmic spherules; even carbonaceous chondrite-derived spherules typically lose nearly all their sulfur through degassing (*26*). Although the SCumPo glasses are clearly depleted relative to the S-rich precursors, the persistence of several weight % S nonetheless indicates that entry heating occurred under reducing conditions that strongly limited oxidation to SO_2_. This suggests that the SCumPo precursors were particularly S-rich particles, resulting in these abnormally reducing conditions upon entry (*25*, *28*). We therefore infer that the lack of magnetite in these spherules is a direct consequence of their C-rich and S-rich nature: Essentially, oxygen was siphoned off by sulfur (and carbon) volatilization, preventing Fe oxidation. This scenario is supported by the presence of immiscible Fe-Ni-S blebs in over half of the particles, as well as by the uniformly Ni-poor olivine compositions. Cosmic spherules that formed under reducing conditions (from metal-poor, C-rich precursors) tend to have NiO-poor neoformed olivine crystals, whereas those formed under more oxidizing conditions (from Ni-bearing metal-rich precursors) can retain higher Ni concentrations in olivine (*11*). The SCumPo spherules fall squarely in the former category: All olivine grains, both relict and neoformed, are extremely low in Ni (fig. S3). In more “normal” Ni-poor cosmic spherules, one still finds magnetite in the mesostasis (*11*), but in SCumPo, the unusually high sulfur content appears to have pushed conditions further toward the reducing end-member. In summary, the mineralogical evidence (metal droplets, Ni-poor olivine, and no magnetite) and geochemical evidence (high retained S) together indicate that SCumPo cosmic spherules formed from a precursor that was both carbon-rich and sulfur-rich, yielding an unusually high reduced state during entry heating.

The oxygen isotope signatures of SCumPo spherules are equally revealing. Nearly all of the data for these spherules lie in the ^16^O-poor field (Fig. 2), reinforcing that they belong to the Group 4 cosmic spherules defined by Suavet *et al.* (*5*). Crucially, however, several analyses targeting relict olivine or mixed relict olivine-glass areas yielded considerably lower δ^18^O and Δ^17^O values, with some even below the TFL. Such values cannot be produced by mass-dependent fractionation of oxygen during atmospheric entry because evaporation-driven isotope shifts move data roughly along the TFL (i.e., they mainly increase δ^18^O, whereas isotopic mixing points toward atmospheric oxygen values near the TFL) (*5*). In other words, a ^16^O-poor silicate melt cannot simply evolve into a ^16^O-rich composition by entry heating; the presence of analyses on both sides of the TFL within one particle must reflect a mixture of two distinct oxygen reservoirs originally present in the particle’s precursor. We interpret this as strong evidence that the SCumPo precursors were composite materials containing at least two different components: one component carrying a ^16^O-rich signature (relatively low δ^18^O and negative Δ^17^O, typical of carbonaceous chondrite anhydrous phases) and another carrying a ^16^O-poor signature (high δ^18^O and positive Δ^17^O not matched to known meteorites but akin to Group 4 fine-grained material). The most straightforward candidates for the ^16^O-rich component are relict forsteritic olivine grains, which could be fragments of chondrules from primitive carbonaceous chondrites (e.g., CM, CO, or related types). The NanoSIMS analysis of the relict olivine core in particle WN-790 yielded δ^18^O ≈ 1.0‰ and Δ^17^O ≈ −3.82‰ (Fig. 2B), which plots along the CM chondrite line and is consistent with olivine from a CM chondrule (*29*). Meanwhile, the ^16^O-poor component is presumably the fine-grained matrix silicate (and any volatiles) that constituted the bulk of the particle; when melted, this matrix yielded the glass and newly crystallized olivine overgrowths with Δ^17^O ~ +2‰. The coexistence of ^16^O-rich relict cores and ^16^O-poor groundmass within the same spherules confirms that the precursor dust grains were heterogeneous in isotopic composition.

This interpretation finds strong support in recent discoveries of large ^16^O-poor unmelted micrometeorites. Suttle *et al.* (*20*, *21*) described three Antarctic micrometeorites (TAM-50-25, TAM-45-133, and TAM-45-140) that have fine-grained, hydrated petrology and bulk oxygen isotopic ratios (δ^18^O ~ 20 to 30‰; Δ^17^O ~ +2‰) similar to Group 4 cosmic spherules. These unmelted particles, proposed as potential precursors of ^16^O-poor cosmic spherules, were found to contain embedded clasts of ^16^O-rich forsteritic olivine (identified as likely chondrule fragments) within their phyllosilicate matrix (*21*). Although destructive analytical techniques prevented measuring the isotopic composition of those individual olivine clasts, their mere presence indicates a mixture of anhydrous chondrule material with a heavily aqueously altered matrix. This is exactly the type of precursor our results suggest for the SCumPo spherules. We conclude that SCumPo cosmic spherules were derived from composite carbonaceous dust particles that contained both primitive chondrule fragments (Fe-poor olivine, carrying a carbonaceous-chondrite-like ^16^O-rich signature) and a fine-grained hydrous matrix (carrying the ^16^O-poor signature). The chondrule fragments survived (at least partially) as relict crystals during entry, whereas the matrix melted and was largely evaporated or oxidized.

Notably, the textures of SCumPo spherules, indicating coexisting μPo and Po domains, also point to a mixed precursor. Van Ginneken *et al.* (*14*) showed that micrometeorite textures reflect precursor grain size and water content: Fine-grained, hydrated precursors tend to produce μPo textures with generally small and frequent vesicles (due to abundant nucleation sites and volatile release), whereas coarse-grained anhydrous precursors yield larger crystals and fewer vesicles. The fact that each SCumPo spherule shows both textures in one particle implies its precursor had a dual nature: a volatile-bearing—possibly sulfur rather than water—fine matrix plus coarse olivine grains. The relict olivines that congregate on one side of the spherule (Fig. 1A) can be explained by their physical settling and displacement during flight (*12*). In essence, the SCumPo particles appear to be the melted remains of the type of composite micrometeorite hypothesized by Suttle *et al.* (*21*), a unique dust from a partially altered parent body that contained chondrule-like inclusions in a hydrated matrix.

Considering potential parent bodies, Suttle *et al.* (*21*) suggested two possibilities for the source of the ^16^O-poor micrometeorites: (i) an unspecified extension of the CM-CO-CY clan, i.e. a primitive carbonaceous body with varying degrees of aqueous alteration, or (ii) a member of the CY (Yamato-type) chondrite group, albeit no known CY chondrites contain substantial phyllosilicate because they are all thermally metamorphosed. Our SCumPo data offer additional clues. We analyzed oxygen isotopes in several relict olivine grains (e.g., in WN-088, WN-349, WN-587, and GMM59), and at least one such analysis (WN-790_S1) yielded a composition clearly consistent with CM chondrule olivine. Note that SIMS data are systematically mixtures of relict cores and neoformed overgrowths, preventing a clear isotopic signature of relict olivine. Nevertheless, this observation lends weight to the hypothesis that a CM-like component is present, favoring the CM-CO-CY clan idea [hypothesis 1 in (*21*)]. It is also noteworthy that the bulk SCumPo compositions plot systematically heavier than CY chondrites, a feature best explained by evaporation-driven mass-dependent fractionation during atmospheric entry; the relatively uniform offset further suggests broadly similar entry-heating conditions, whereas abundant sulfides in the precursors would have minimized equilibration with atmospheric O_2_, reinforcing the dominance of evaporation in shaping their isotopic signatures. We also note that CY chondrites are exceptionally rich in sulfides [~10 to 30 vol % troilite (*30*)], which is consistent with the high sulfur content observed in SCumPo spherules. A CY-like body would readily provide the S needed to suppress magnetite formation. It is possible that the true parent material is an intermediate or transitional type between known CM/CO and CY chondrites, essentially a recently identified branch of carbonaceous chondrite. On the basis of our results, we propose that SCumPo cosmic spherules (and by extension most Group 4 cosmic spherules) are linked to the CM-CO-CY chondrite clan and represent an extension of this clan to a highly primitive but sulfide-rich end-member. In other words, these micrometeorites likely sample a parent body that has not yet been identified in meteorite collections: a carbonaceous asteroid (or cometary fragment) that experienced considerable aqueous alteration (like CM/CO chondrites) but retained abundant Fe-sulfides (like CY chondrites). This parent body would produce fine-grained, ^16^O-poor dust with embedded ^16^O-rich olivine, exactly the type of precursors required for SCumPo cosmic spherules.

### Orbital parameters of the precursor particles and implications for the identification of their parent body

Numerical modeling of the settling of olivine within S-type cosmic spherules was performed following the methods of Genge *et al.* (*12*) to investigate the entry velocities required to cause sufficient settling to produce cumulate textures. Genge *et al.* (*12*) examined ordinary chondrite precursors and found that olivine phenocrysts could not settle effectively during atmospheric deceleration because they grow during cooling after peak deceleration has been experienced. Genge *et al.* (*12*) found, however, that relict forsterite (Mg-rich) olivine that survives melting can settle in particles with higher entry velocities (>16 km/s) and act as nucleation centers for the growth of olivine phenocrysts to produce a cumulate texture.

Simulations of CI and CY chondrite precursors were performed here using premelting densities of 2.4 and 2.5 g cm^−3^, respectively, with increases in density occurring during melting as the pore space is destroyed. Settling simulations of 20-μm-diameter phenocrysts grown from melt and 10-μm-diameter relict forsterites were undertaken to investigate the formation of cumulate textures in particles of CI and CY chondrite composition. Settling of sulfide liquid droplets was also undertaken to examine whether these would separate from silicates under the conditions in which cumulate olivine formation occurred.

**Fig. 3.**
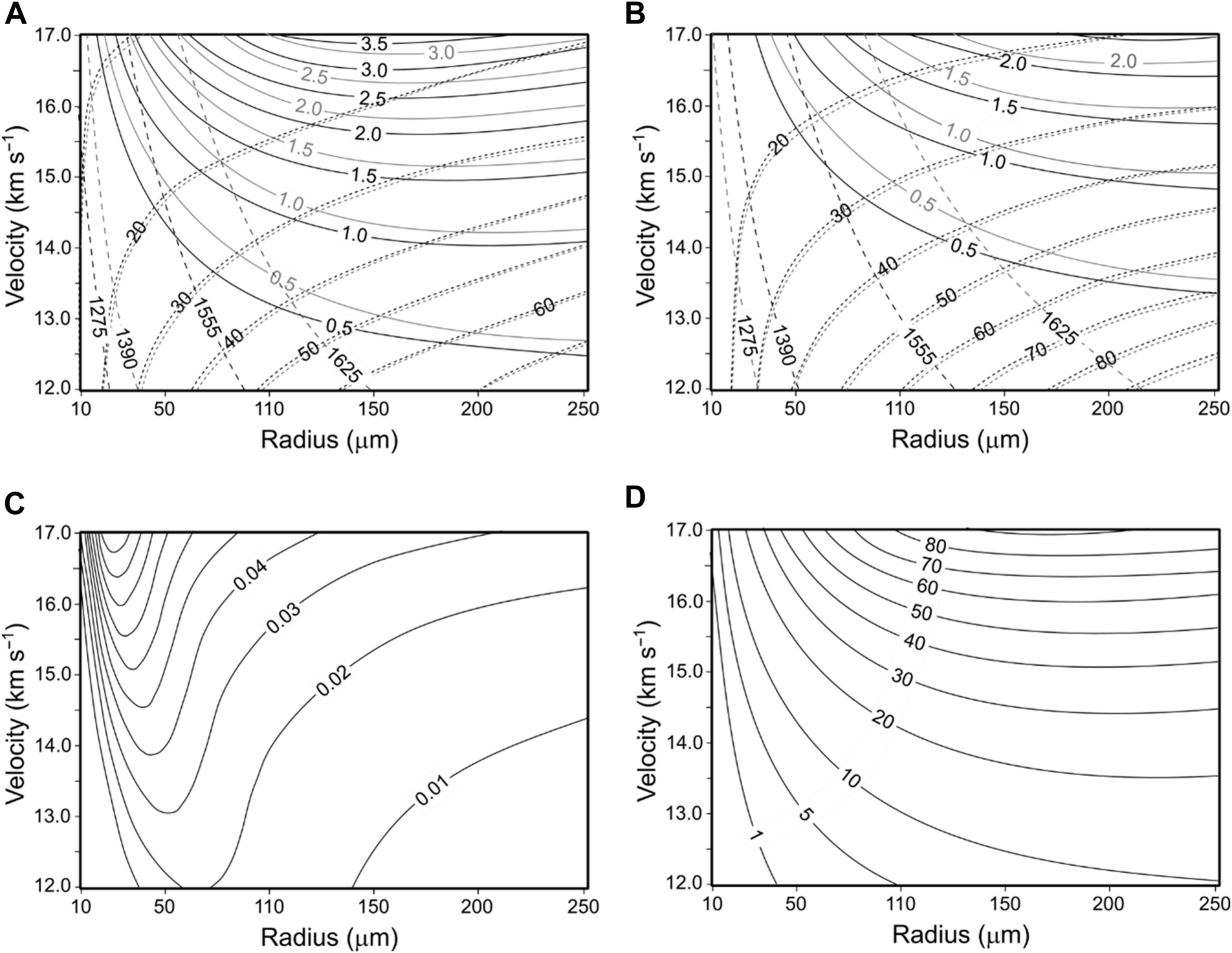
Settling simulation results. Solid lines show the settling distance relative to particle radius (pr), dashed lines show isotherms for 50% crystals (CI: 1275°C; CY: 1390°C) and the liquidus (CI: 1555°C; CY: 1625°C), and dotted lines show the final particle radius. Black lines are for CI chondrites, and gray lines are for CY chondrites. (**A**) Settling of relict forsterite at a 90° entry angle. (**B**) Settling of relict forsterite at a 45° entry angle. (**C**) Settling of olivine phenocrysts at a 90° entry angle. (**D**) Settling of sulfide liquids (constant density of 4.75 g cm^−3^).

Simulation results suggest that the settling distances of relict forsterites are smaller in CI and CY (Fig. 3) than in the case of the ordinary chondrite precursors modeled by Genge *et al.* (*12*) owing to greater deceleration before melting and the higher liquidus temperatures (~1625°C compared to 1440°C), which lead to higher effective melt viscosities owing to larger crystal contents. Genge *et al.* (*12*) also used a constant density for forsterite relicts, whereas here their temperature-dependent density is calculated (for a composition of Fo_98_), resulting in lower values for crystal density and thus decreased settling. Assuming a settling distance threshold equal to particle radius (1.0 pr) for the formation of cumulate textures, a minimum entry velocity of ~16.1 and 15.2 km s^−1^ is predicted for the onset of cumulate textures within CI and CY precursors, respectively (Fig. 3C). Cumulate texture formation starts at entry angles of 90° to the horizontal at which deceleration is maximized. The entry velocities required for the average particle (entry angle of 45°) are higher at ~17.2 and 16.1 km s^−1^ (Fig. 3B). Similar to the observations of Genge *et al.* (*12*), phenocrysts grown during settling do not experience notable settling (Fig. 3C). Simulations of sulfide settling suggest rapid separation of liquid sulfide from silicates under these entry parameters (Fig. 3D).

If SCumPo spherules form at subliquidus temperatures, the simulations reported here would suggest very small maximum final diameters of 20 to 40 μm. In contrast, observed SCumPos have an average diameter of 127 μm, with a maximum diameter of 211 μm. SCumPo spherules could, however, form at higher peak temperatures because forsterite relicts have one-component melting temperatures of 1890°C and as relicts indicate nonequilibrium melting. Although the equilibrium liquidus of the bulk silicate system is typically reached at ~1450° to 1550°C, the complete dissolution of Mg-rich olivine is a diffusion-limited process that requires considerably more time than the 1 to 2 s of peak heating experienced during entry (*12*). Furthermore, intense evaporative mass loss at temperatures above 1700°C acts as a thermal buffer, preventing particles from reaching the kinetic melting point of pure forsterite (2063 K) (*31*). Phenocrysts can then form on cooling by nucleation on these surviving relict forsterites. This preservation of solid nuclei is essential as it prevents the transition to a cryptocrystalline or glassy texture that would otherwise result from the cooling of a total, superheated melt (*14*). The formation of SCumPo spherules at supraliquidus temperatures allows larger SCumPo particles to form, particularly at low entry angles. The entry velocities required at larger sizes tend toward 14.2 and 14.5 km s^−1^ (for CI and CY compositions, respectively) at an entry angle of 90° and 15.0 and 15.2 km s^−1^ at an entry angle of 45° (Fig. 3, A and B).

Last, the effects of vesicles were not considered within the current numerical models but warrant consideration. Genge *et al.* (*32*) showed that expansion particles owing to the vesiculation of particles on melting results in decreases in peak temperature but that vesicles are rapidly lost with increasing degree of partial melting as a result of deceleration. Most SCumPo spherules have low vesicle contents compared to other porphyritic spherules, consistent with higher decelerations and peak temperatures. However, because the settling of relict crystals occurs during the heating phase, when deceleration is higher, the presence of vesicles before their separation is likely to impede settling owing to both the adhesion of relict crystals to vesicles by surface tension and the increase in viscosity caused by vesicularity. To enable settling of relicts, the precursors of SCumPo particles may have to be anhydrous because Genge *et al.* (*32*) suggested that water vapor dominates vesiculation at low temperatures, with oxidation of sulfides becoming more important at high temperatures. This constraint suggests that CI-like particles are unlikely to form SCumPo particles and, consequently, CY-like materials, which have already experienced parent body dehydration [typically retaining ~5 wt % water (*30*)], are more likely precursors, in particular because their sulfide components very rapidly separate to the leading face of the particle and mostly degas directly into the atmosphere.

Entry velocity into Earth’s atmosphere is dependent on encounter velocity and thus eccentricity and semimajor axis of the orbit at the point of capture (*12*). Although no one value of the parameters exists, most solutions for SCumPos suggest orbits with eccentricities of >0.26 at Earth encounter for a minimum velocity of 14.2 km s^−1^.

### A near-Earth source for ^16^O-poor cosmic spherules

The high entry velocities inferred for SCumPo precursors have implications for their orbital parameters. Encounter speeds of 14 to 17 km s^−1^ at Earth typically correspond to particles on orbits with considerable eccentricity relative to Earth’s orbit. Using the relations described by Genge *et al.* (*12*), an entry velocity of ~14 km s^−1^ suggests that the dust particle was on an orbit with eccentricity *e* > 0.2 when it hit Earth. Such orbits can be indicative of Jupiter-family comets or high-eccentricity near-Earth asteroids [near-Earth objects (NEOs)], rather than main-belt asteroids, which produce dust with *e* < 0.2 (*33*). Most cosmic spherules collected on Earth are thought to originate from main-belt asteroidal sources with low eccentricities; however, the SCumPo spherules appear to require a different source. Given that we have linked their composition to the CM-CO-CY clan of carbonaceous chondrites, it is plausible that their parent body was a primitive carbonaceous asteroid that migrated onto an Earth-crossing orbit—perhaps through resonance or planetary perturbation—attaining comet-like orbital parameters. For instance, one might consider the disrupted fragments of a thermally altered but water-bearing asteroid (like the CY group) that evolved into near-Earth space. The high eccentricity of SCumPo’s suggests that dust production occurs while the parent body is in the NEO population and enters Earth’s atmosphere at high velocity, and the dust’s composition would be characterized by a mix of anhydrous and hydrous phases with ^16^O-poor isotopic signature.

One such candidate is (3200) Phaethon, a NEO associated with the Geminid meteor shower. Recent studies have proposed Phaethon as a likely parent body of CY chondrites, based on both its spectral properties and its volatile emission behavior under rapid heating, which suggest that it is an S-rich body—either CY or CY-like (*34*, *35*). However, Geminid meteoroids typically enter Earth’s atmosphere at ~35 km s^−1^, a velocity far too high for dust-sized particles to survive atmospheric entry (*31*). This makes it unlikely that SCumPo spherules, which preserve relict phases and ^16^O-poor isotopic anomalies, are derived from Geminid-type dust.

Our findings thus point to a candidate parent—a primitive C-type near-Earth asteroid related to the CY chondrites—as the long-lasting source of ^16^O-poor cosmic spherules found in both recent urban deposits (*36*) and ~3-million-year-old Antarctic sediments (*21*). This hypothetical parent body would effectively represent a “missing” meteorite type—sampled by micrometeorites but not yet in our meteorite collections—and its identification in future asteroid missions or meteorite finds would be a pivotal discovery.

## MATERIALS AND METHODS

The micrometeorites studied here were selected from the SRM, LK, and BU micrometeorite collections. Details on the geological contexts and sampling protocols of these collections can be found in (*15*, *22*, *23*, *37*, *38*).

### Sample preparation and electron microscopy

Each cosmic spherule was embedded in epoxy and polished for analysis. BSE images, and quantitative major- and minor-element compositions of WN and LK samples were obtained using a JEOL JXA-8200 electron probe microanalyzer (EPMA) at the National Institute of Polar Research (Tokyo, Japan). Analyses were performed on both sulfide phases and silicate/oxide phases within the SCumPo cosmic spherules. For sulfide phases, we used a focused beam of <1 μm in diameter at a 30-nA beam current, analyzing the elements Mg, Al, Si, P, S, Cr, Fe, Co, Ni, Cu, and Zn. For silicate and oxide phases (olivine, magnetite, glass, etc.), we used a focused <1-μm beam at 10 nA, monitoring Na, Mg, Al, Si, P, S, Cl, K, Ca, Ti, V, Cr, Mn, Fe, Co, and Ni. In addition, a defocused 20-μm beam at 10 nA was used to acquire bulk-average compositions of glassy regions (spherule mesostasis). The accelerating voltage was 15 kV for all analyses. Counting times on peak were 10 to 100 s per element, with ZAF corrections applied. Interelement interference corrections were made for V-Ti, Co-Fe, and Cu-Ni overlaps. A suite of natural and synthetic mineral and metal standards was used for calibration.

The GMM samples were analyzed at Utrecht University using a JEOL JXA-8530F field-emission electron microprobe. Operating conditions were a 40° take-off angle, 20-kV accelerating voltage, 15-nA beam current, and beam diameters between 1 and 10 μm. Elements were measured with appropriate crystals (LIFH for Fe, Mn, Ni, Cr, and Ti; PETH for Ca, K, and S; TAPH for Mg and Na; TAP for Si, P, and Al). Counting times were 20 to 40 s with matched off-peak durations, and backgrounds were fitted using linear or exponential models depending on the element. Calibration used a suite of natural and synthetic standards (diopside, jadeite, pyrite, KTiPO_5_, nickel, chromium, tephroite, GOR-132, KL-2, and MongOl-SH11-2). Deadtime and interference corrections were applied, and oxygen was calculated by cation stoichiometry and included in the matrix corrections.

Analytical detection limits for key elements (e.g., S, Ni, and Co) are listed in tables S2 to S5. Uncertainties (1σ) for major elements are typically <5% relative.

### Oxygen isotope analyses (SIMS)

In situ oxygen three-isotope measurements were carried out with a CAMECA IMS 1270 E7 secondary ion mass spectrometer at CRPG-CNRS (Nancy, France). An ~2.5-nA Cs^+^ primary beam was focused to ~15 μm in diameter. Secondary ions of O^−^ were measured in multicollection mode: ^16^O^−^ and ^18^O^−^ were detected on two off-axis Faraday cups, whereas ^17^O^−^ was measured on the axial Faraday cup. Instrumental mass resolving power (MRP) was adjusted to ~7000 (at *M*/Δ*M* for ^17^O) using entrance/exit slits to eliminate the ^16^OH^−^ interference on ^17^O and to ensure flat-topped peaks for ^16^O and ^18^O. The secondary magnet was cycled between two positions to alternately measure the ^16^O and ^18^O on the off-axis cups and the ^17^O on-axis cups, with appropriate background corrections. Each analysis consisted of a 90-s presputter to remove the gold coating and achieve sputter equilibrium, followed by 180 s of data acquisition. Five in-house mineral standards (San Carlos olivine, CLDR01 MORB glass, Burma spinel, “Gold” enstatite, and JV1 diopside) were measured repeatedly to define the TFL and to correct instrumental mass fractionation (IMF) and any matrix-dependent bias. Typical secondary count rates on San Carlos olivine were ~1.3 × 10^9^ cps for ^16^O, ~5 × 10^5^ cps for ^17^O, and ~2.5 × 10^6^ cps for ^18^O. Reproducibility (2σ) on the standards was ±0.4‰ for δ^18^O, ±0.5‰ for δ^17^O, and ±0.6‰ for Δ^17^O (where Δ^17^O is the deviation from the TFL, defined as Δ^17^O = δ^17^O − 0.52 × δ^18^O).

### Oxygen isotope analyses (NanoSIMS)

High-spatial-resolution in situ O-isotope analyses of sub–10-μm relict olivines in four SCumPo spherules were performed with a CAMECA NanoSIMS 50L at The Open University (OU). Because of the small sizes of olivine relicts and the presence of nearby phases (glass or voids), particular care was taken in placing the analysis spots; BSE and secondary electron images were used to target the approximate grain centers. Before analysis, the sample area was presputtered with a focused 16-kV ~100-pA Cs^+^ probe for 2 min over an area of 5 μm by 5 μm to remove the carbon coating, possible surface contamination, and to achieve sputter equilibrium. Analyses were undertaken using a focused 16-kV ~80-pA Cs^+^ probe (<0.5 μm in diameter) that was rastered over an area of 3 μm by 3 μm in “spot” mode. An electron flood gun was used for charge compensation. Seven different secondary ion species were collected simultaneously. ^16^O^−^ was measured on a Faraday detector, whereas ^17^O^−^, ^18^O^−^, ^30^Si^−^, ^26^Mg^16^O^−^, ^40^Ca^16^O^−^, and ^56^Fe^16^O^−^ were measured using electron multipliers. An MRP of ~10,000 (Cameca definition, based on the measured peak width between 10 and 90% of the peak) was used, which is sufficient to resolve the ^16^OH^−^ interference from the ^17^O^−^ signal. Analyses lasted for ~7 min, providing a total of ~1.5 × 10^10^ counts ^16^O^−^. Multiple small grains of San Carlos olivine (δ^18^O 4.91‰ as measured by laser fluorination at the OU) were mounted alongside samples as the standard for IMF and Δ^17^O reference. Analytical uncertainty (2σ), incorporating internal counting statistics from the sample measurement and external precision from standard replicates analyzed before and/or after the samples, is typically ±1.2‰ for δ^17^O, ±0.9‰ for δ^18^O, and ~±0.8‰ for Δ^17^O. Matrix effects were corrected using San Carlos olivine (Fo_90_), Eagle Station pallasite (Fo_80_), and an olivine with a composition of Fo_72_ and the method described by Zhang *et al.* (*39*) to account for differences in the Fe/Mg of the samples of olivine to the standard. The location of each raster pit, as well as the absence of any notable cracks or inclusions, was verified using SEM following analyses to remove any compromised analyses.

### Numerical model

Settling of crystals within cosmic spherules during atmospheric entry involved numerical integration of the partial differential equations of motion and thermal behavior of extraterrestrial dust (*12*). Mass loss through evaporation was achieved using a Langmuir expression for the evaporation of silicate materials (*12*, *31*). Although evaporation rates are likely to vary with silicate composition, these simulations focus on porphyritic cosmic spherules that have experienced peak temperatures below the liquidus where partial evaporation effects, and compositional change, are minimal. Simulations of particles with entry angles of 90° to the horizontal were performed because these particles experience the highest decelerations and thus represent the minimum entry velocities at which settling can occur.

The settling distance of crystals was calculated using Stokes law at each timestep and assumes silicate liquids are Newtonian. The effect of crystals on viscosity was modeled using the Einstein-Roscoe equation and are inversely proportional to melt volume. Bulk melts of chondritic materials are ultrabasic magmas with very low yield strengths and thus closely approximate to Newtonian fluids. Increases in silica content of residual liquids during crystallization, however, increases yield strength. The high crystal abundance once residual liquids have fractionated to higher silica content prevents appreciable settling; thus, non-Newtonian behavior of the fluid has a limited influence on the cumulative displacement of crystals. Note that the current numerical models do not account for aerodynamic rotation during atmospheric flight. Although substantial rotation could modify simple settling geometries by driving phases outward, the high-density contrast between Fe-Ni-S droplets and silicate liquid suggests that dense phases would still effectively migrate toward the leading hemisphere under the decelerations modeled here. Furthermore, although thermal gradients likely exist within particles during entry heating, the brief duration of peak temperatures and the effects of evaporative cooling minimize the impact of these gradients on the overall survival of relict phases.

The abundances and phase compositions for CI and CY chondrite compositions were determined through thermodynamic modeling using pmelts version 1.02 (*40*). The bulk compositions of the meteorites Y-86720 and Ivuna were used for CY and CI chondrites, respectively (table S6) (*30*). The melt was modeled as anhydrous owing to the very low solubility of silicate melts for water at low pressures. Thermodynamic calculations were performed to determine the equilibrium state of the system at temperature intervals of 5°C, and linear interpolation was performed to allow an estimate of these values at any temperature. Thermodynamic equilibrium is unlikely to be attained during rapid cooling during atmospheric entry. Observations of S-type cosmic spherules indicate that pyroxene and plagioclase crystallization are kinetically impeded during cooling; thus, these phases were suppressed in the thermodynamical calculation. Supercooling of olivine or spinel was not, however, included because both phases are present in S-type cosmic spherules. In CumPo spherules, olivine crystals are usually zoned, indicating disequilibrium crystallization. Melt compositions from equilibrium calculations are thus likely to underestimate the iron content within the melt, but this has a second-order effect on melt viscosity. Olivine phenocrysts in CumPo spherules are equant rather than skeletal and thus testify to minimal supercooling presumably because these contain abundant heterogeneous nuclei because peak temperatures are subliquidus. Phase compositions were used to calculate melt viscosity (fig. S4) using expressions from Giordano *et al.* (*41*) and phase densities after Niu and Batiza (*42*).

A major difference to the settling calculations performed by Genge *et al.* (*12*), which focused on ordinary chondrite precursors, was the treatment of particle density before melting. Ordinary chondrites experience only small changes in density on melting, whereas CI and CY chondrites experience substantial increases in density on melting as the pore space is destroyed. A density of 2.4 g cm^−3^ was used for unmelted CI chondrite, whereas 2.5 g cm^−3^ was used for CY chondrite precursors. Density change owing to melting was modeled as a smooth step function over a temperature interval of 50°C above the solidus temperature to approximate destruction of the pore space. The effect of the lower density of CI and CY precursors increases deceleration before melting compared to ordinary chondrites and decreases the peak temperature attained. Higher entry velocities are thus required to cause crystal settling.
