# Supplementary material for: 16O poor cosmic spherules from near-Earth CY chondrite asteroids
Source: Sci Adv. 2026 Jun 26;12(26):eaed6340. doi: 10.1126/sciadv.aed6340 (PMC13308587; doi:10.1126/sciadv.aed6340)
Supplement: Supplementary file 1 — Figs. S1 to S4 Tables S1 to S6 [file sciadv.aed6340_sm.pdf]

Science Advances
AAAS

Supplementary Materials for

^{16}O poor cosmic spherules from near-Earth CY chondrite asteroids

Matthias Van Ginneken *et al.*

Corresponding author: Matthias Van Ginneken, m.van-ginneken@kent.ac.uk

Sci. Adv. **12**, eaed6340 (2026)
DOI: 10.1126/sciadv.aed6340

This PDF file includes:

Figs. S1 to S4
Tables S1 to S6

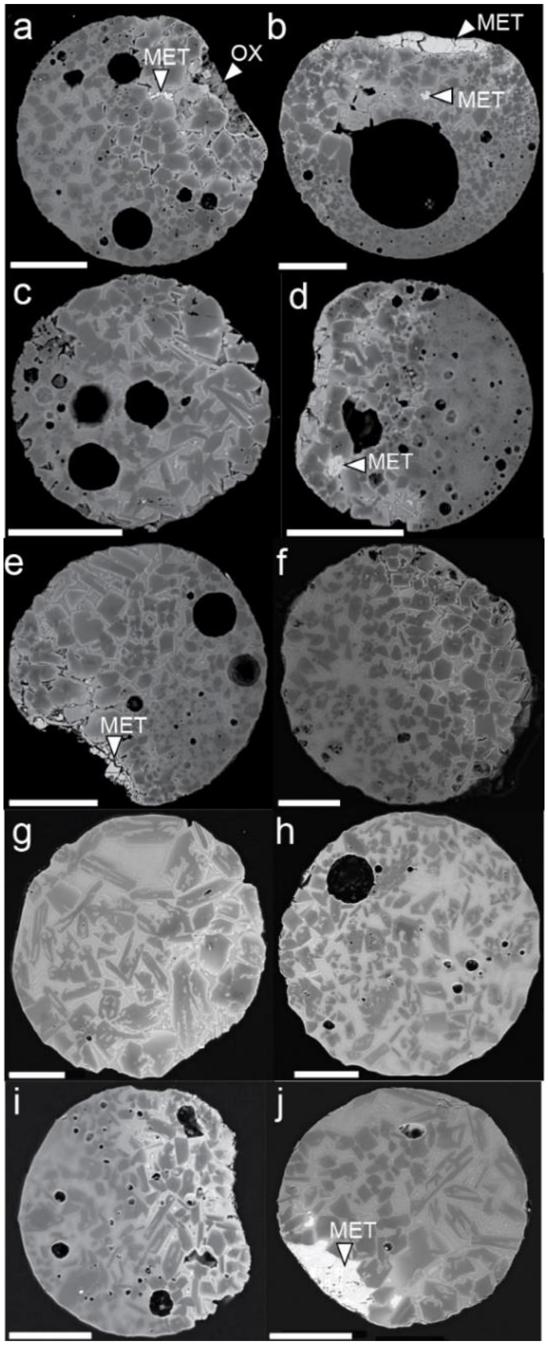

Fig.S1. **Backscattered electron images of sections of the cosmic spherules studied.** Particles WN-008 (**A**), WN-349 (**B**), WN-464 (**C**), WN-587 (**D**) and WN-790 (**E**) from the Walnumfjellet collection; LK06-0395 (**F**) from the Larkman Nunatak collection; and GMM-59 (**G**), GMM-75 (**H**), GMM-136 (**I**) and GMM-422 (**J**) from the Budel collection. Visible Fe-Ni-S metal is indicated (MET). Particle WN-088 exhibits oxyhydroxides (OX) as corrosion products of metal bead due to terrestrial weathering (*43*). Scalebar is 50 µm. Consistent with Figure 1, the plane of section may not necessarily intersect the true aerodynamic axis of the spherule. Consequently, the apparent prominence of the cumulate texture and the relative distribution of dense phases are highly dependent on the orientation of the section.

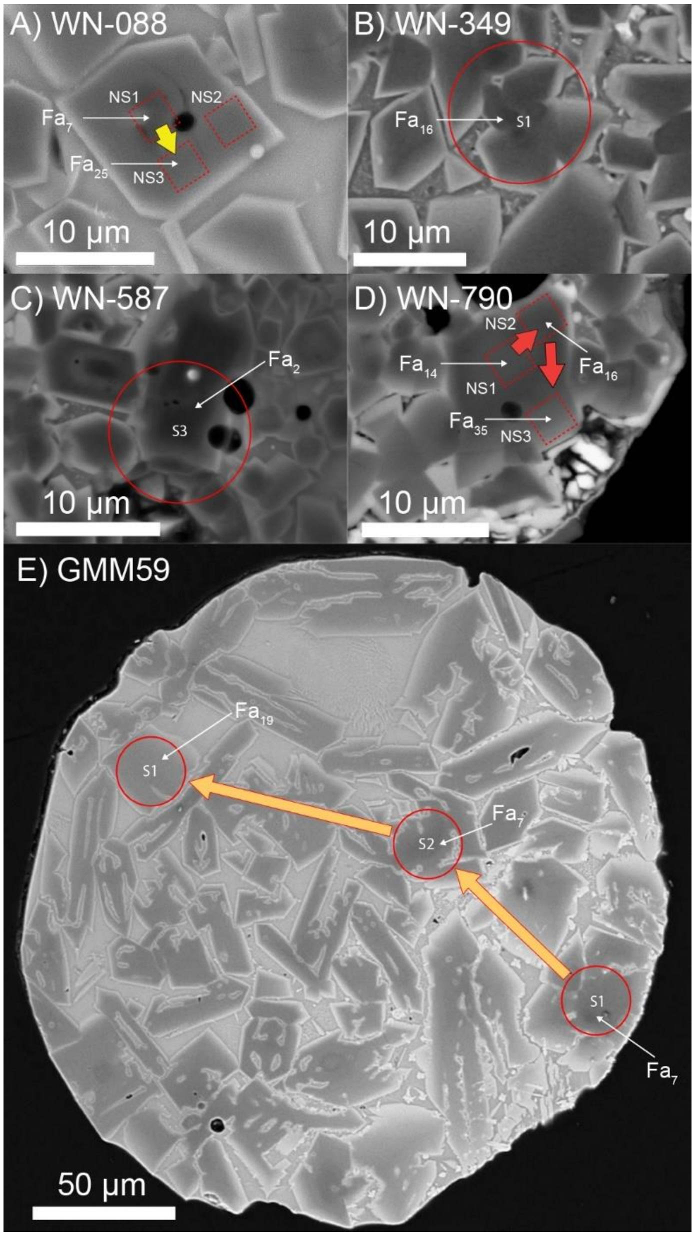

Fig.S2. **Backscattered electron images of relict olivine phenocrysts in SCumPo cosmic spherule GMM-59.** Red dashed squares and red circles represent NanoSIMS and SIMS analysis locations, respectively. Datapoint labels correspond to data in Table S1. Coloured arrows match those in fig.2.

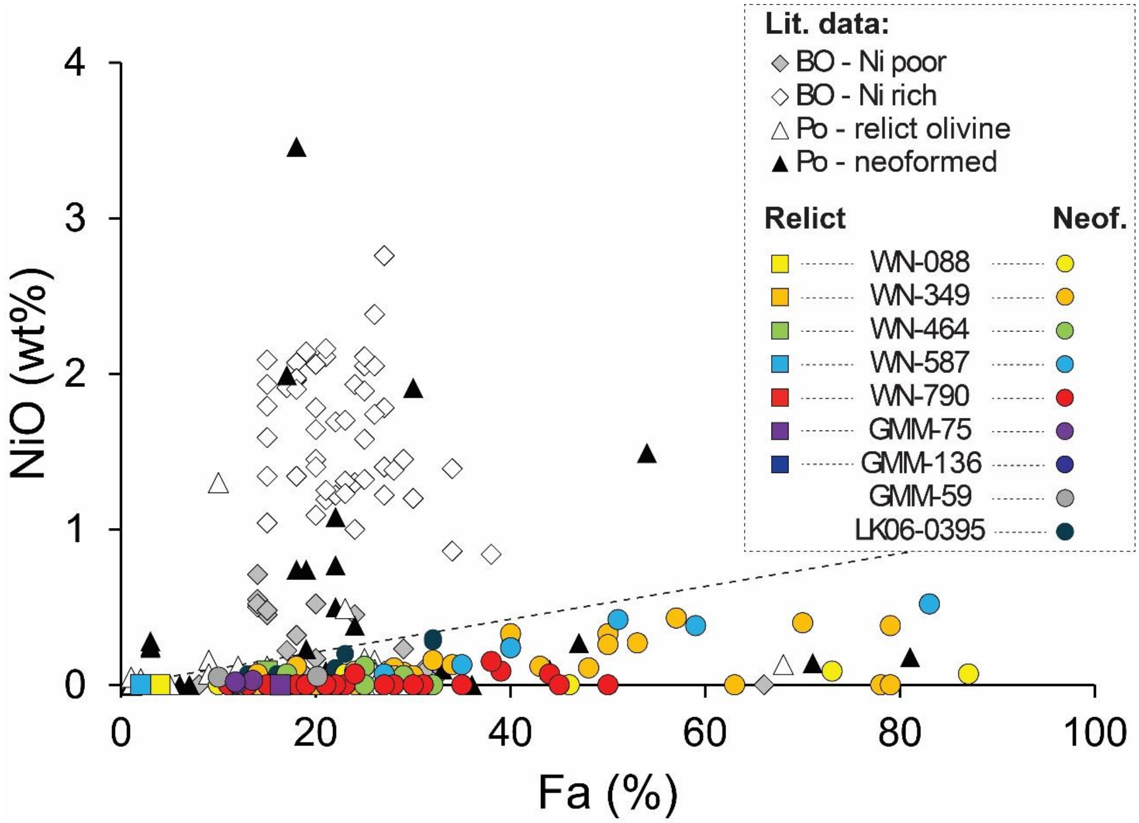

Fig.S3. **Plot of fayalite content vs. NiO (wt%) concentration in cosmic spherules**. Olivine composition in SCumPo cosmic spherules is compared to relict and neoformed olivine in barred olivine (BO) and porphyritic olivine (Po) cosmic spherules (*11*).

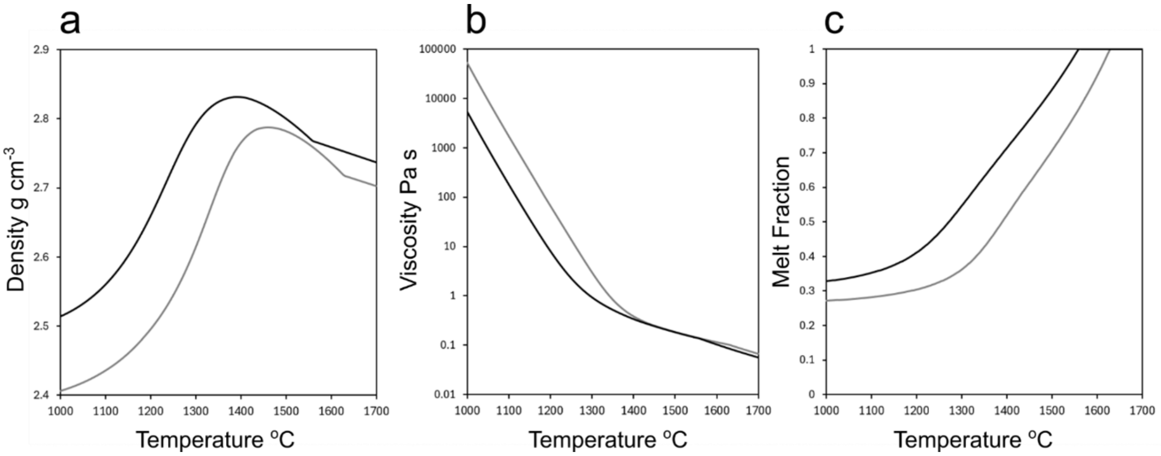

Fig.S4. **The properties of melt predicted from thermodynamic calculations of the bulk composition.** (**A**) Density, (**B**) viscosity, (**C**) melt fraction. Black is CI chondrite, grey is CY chondrite.

Table S1. **Oxygen isotopic compositions SCumPo cosmic spherules.**

Analysis#	$\delta^{17}O$	2σ	$\delta^{18}O$	2σ	$\Delta^{17}O$	2σ	*Target material*
Secondary-ion mass spectrometry measurements							
**WN-007**							
WN-007_S1	0.62	0.4	4.2	0.41	-1.57	0.5	Neof. Olivine/Glass
WN-007_S2	-5.96	0.5	-3.6	0.49	-4.10	0.5	Rel. Olivine
WN-007_S3	12.87	0.5	27.7	0.44	-1.52	0.5	Neof. Olivine/Glass
**WN-033**							
WN-033_S1	12.5	0.4	27.2	0.43	-1.66	0.5	Neof. Olivine/Glass
**WN-088**							
WN-088_S1	23.8	0.5	41.6	0.3	2.16	0.5	Neof. Olivine/Glass
WN-088_S2	22.4	0.4	39.2	0.4	1.99	0.4	Neof. Olivine/Glass
WN-088_S3	23.3	0.4	42.5	0.4	1.16	0.5	Neof. Olivine/Glass
**WN-349**							
WN-349_S1	12.4	0.7	26.5	0.5	-1.43	0.7	Relict Olivine/Glass
WN-349_S2	23.4	0.5	41.3	0.3	1.96	0.5	Neof. Olivine/Glass
WN-349_S3	23.7	0.4	40.9	0.3	2.41	0.4	Neof. Olivine/Glass
WN-349_S4	22.3	0.4	40.8	0.4	1.11	0.5	Neof. Olivine/Glass
WN-349_S5	23.2	0.4	40.9	0.4	1.91	0.4	Neof. Olivine/Glass
**WN-464**							
WN-464_S1	20.7	0.7	39.5	0.4	0.17	0.7	Neof. Olivine/Glass
WN-464_S2	19.9	0.6	36.7	0.5	0.83	0.6	Neof. Olivine/Glass
WN-464_S3	22.4	0.6	38.9	0.4	2.14	0.6	Neof. Olivine/Glass
WN-464_S4	20.4	0.5	35.8	0.5	1.83	0.5	Neof. Olivine/Glass
WN-464_S5	20.4	0.6	38.4	0.4	0.42	0.7	Neof. Olivine/Glass
WN-464_S6	22.4	0.5	40.3	0.4	1.44	0.6	Neof. Olivine/Glass
**WN-587**							
WN-587_S1	22.3	0.5	38.5	0.3	2.24	0.5	Neof. Olivine/Glass
WN-587_S2	24.6	0.5	42.7	0.3	2.45	0.6	Neof. Olivine/Glass
WN-587_S3	10.6	0.9	24.0	1.0	-1.88	1.0	Relict Olivine/Glass
**WN-790**							
WN_790_S1	22.4	0.4	40.7	0.4	1.23	0.4	Neof. Olivine/Glass
WN_790_S2	21.4	0.5	37.1	0.4	2.10	0.6	Neof. Olivine/Glass
WN_790_S3	24.2	0.4	42.5	0.4	2.09	0.5	Neof. Olivine/Glass
WN_790_S4	23.7	0.5	41.4	0.3	2.17	0.5	Neof. Olivine/Glass
WN_790_S5	22.8	0.4	40.1	0.3	1.91	0.5	Neof. Olivine/Glass
**GMM59**							
GMM59_S1	17.1	0.3	31.8	0.3	0.61	0.4	Neof. Olivine
GMM59_S2	11.8	0.4	22.9	0.6	-0.09	0.5	Neof./Rel. Olivine
GMM59_S3	2.7	0.5	8.6	0.5	-1.81	0.5	Neof./Rel. Olivine
GMM59_S4	20.5	0.3	37.3	0.3	1.10	0.4	Neof. Olivine
GMM59_S5	19.7	0.4	35.5	0.3	1.19	0.4	Neof. Olivine
GMM59_S6	21.0	0.4	38.5	0.3	0.92	0.4	Neof. Olivine

Table S1. continued

Analysis#	***$\delta^{17}O$***	***2σ***	***$\delta^{18}O$***	***2σ***	***$\Delta^{17}O$***	***2σ***	***Target material***
**GMM75**							
GMM75_S1	37.3	0.3	20.5	0.3	1.10	0.4	Neof. Olivine/Glass
GMM75_S2	35.5	0.3	19.7	0.4	1.19	0.4	Neof. Olivine/Glass
GMM75_S3	38.5	0.3	21.0	0.4	0.92	0.4	Neof. Olivine/Glass
**GMM136**							
GMM136_S1	18.7	0.4	32.3	0.4	1.87	0.4	Neof. Olivine
GMM136_S2	13.2	0.4	23.9	0.4	0.76	0.4	Neof./Rel. Olivine
GMM136_S3	13.4	0.4	22.8	0.6	1.54	0.5	Neof./Rel. Olivine
**GMM422**							
GMM422_S1	23.4	0.6	41.5	0.4	1.80	0.6	Neof. Olivine/Glass
GMM422_S2	23.9	0.6	42.6	0.4	1.81	0.6	Neof. Olivine/Glass
GMM422_S3	24.3	0.6	42.8	0.4	2.09	0.6	Neof. Olivine/Glass
<u>*Nanoscale secondary-ion mass spectrometry measurements*</u>							
**LK06-0395**							
LK06-0395_NS1	25.7	1.2	46.1	0.7	1.73	0.8	Neof. Olivine
LK06-0395_NS2	21.0	1.2	39.0	0.7	0.68	0.8	Neof. Olivine
LK06-0395_NS3	24.1	1.2	43.4	0.7	1.48	0.8	Neof. Olivine
LK06-0395_NS4	22.0	1.1	40.3	0.7	1.01	0.8	Neof. Olivine
LK06-0395_NS5	24.3	1.1	42.9	0.7	2.02	0.8	Neof. Olivine
LK06-0395_NS6	23.0	1.2	41.5	0.7	1.40	0.8	Neof. Olivine
LK06-0395_NS7	24.1	1.2	43.1	0.7	1.71	0.8	Neof. Olivine
LK06-0395_NS8	23.6	1.2	40.7	0.7	2.44	0.8	Neof. Olivine
LK06-0395_NS9	25.3	1.2	44.5	0.7	2.13	0.8	Neof. Olivine
LK06-0395_NS10	24.1	1.1	45.6	0.7	0.36	0.8	Neof. Olivine
LK06-0395_NS11	24.9	1.0	45.1	0.7	1.45	0.7	Neof. Olivine
LK06-0395_NS12	21.4	1.0	37.6	0.7	1.88	0.7	Neof. Olivine
LK06-0395_NS13	23.4	1.1	41.4	0.7	1.88	0.7	Neof. Olivine
LK06-0395_NS14	21.6	1.1	37.5	0.7	2.15	0.7	Neof. Olivine
LK06-0395_NS15	24.1	0.9	41.1	0.7	2.78	0.7	Neof. Olivine
LK06-0395_NS16	20.9	0.8	35.7	0.7	2.37	0.7	Neof. Olivine
**WN-088**							
WN-088_NS1	14.1	1.0	28.8	0.7	-0.91	0.8	Rel. Olivine
WN-088_NS2	23.8	1.0	41.0	0.7	2.47	0.8	Neof. Olivine
WN-088_NS3	26.5	1.1	45.3	0.7	2.98	0.8	Neof. Olivine
WN-088_NS4	26.8	1.0	45.9	0.7	2.97	0.8	Neof. Olivine
WN-088_NS5	24.3	1.0	44.4	0.7	1.22	0.8	Neof. Olivine
WN-088_NS6	22.8	1.0	39.2	0.7	2.47	0.8	Neof. Olivine
**WN-349**							
WN-349_NS1	25.3	1.2	40.7	0.9	4.13	0.7	Neof. Olivine
WN-349_NS2	26.5	1.2	44.4	0.9	3.35	0.7	Neof. Olivine
WN-349_NS3	28.1	1.2	47.8	0.9	3.25	0.7	Neof. Olivine
WN-349_NS4	22.3	1.2	40.3	0.9	1.30	0.7	Neof. Olivine
WN-349_NS5	26.5	1.1	46.7	0.9	2.22	0.7	Neof. Olivine

Table S1. continued

Analysis#	***$\delta^{17}O$***	***2σ***	***$\delta^{18}O$***	***2σ***	***$\Delta^{17}O$***	***2σ***	***Target material***
WN-349_NS6	24.9	1.2	43.0	0.9	2.49	0.7	Neof. Olivine
WN-349_NS7	26.0	1.2	43.4	0.9	3.42	0.7	Neof. Olivine
WN-349_NS8	23.2	1.1	41.1	0.9	1.77	0.7	Neof. Olivine
WN-349_NS9	24.7	1.1	42.1	0.9	2.81	0.7	Neof. Olivine
WN-349_NS10	30.3	1.3	51.3	0.9	3.62	0.7	Neof. Olivine
WN-349_NS11	28.2	1.4	51.2	1.0	1.55	0.7	Neof. Olivine
WN-349_NS12	28.4	1.3	50.0	0.9	2.44	0.7	Neof. Olivine
WN-349_NS13	23.5	1.2	38.6	0.9	3.43	0.7	Neof. Olivine
WN-349_NS14	23.2	1.2	40.5	0.9	2.12	0.7	Neof. Olivine
WN-349_NS15	24.0	1.2	41.5	0.9	2.45	0.7	Neof. Olivine
WN-349_NS16	22.9	1.2	39.8	0.9	2.14	0.7	Neof. Olivine
WN-349_NS17	22.4	1.2	37.3	0.9	3.00	0.7	Neof. Olivine
WN-349_NS18	28.8	1.2	49.6	0.9	3.01	0.7	Neof. Olivine
WN-349_NS19	27.5	1.3	46.9	0.9	3.10	0.7	Neof. Olivine
WN-349_NS20	30.6	1.3	54.3	1.0	2.35	0.7	Neof. Olivine
**WN-790**							
WN-790_NS1	-3.3	1.0	1.0	0.7	-3.82	0.5	Rel. Olivine
WN-790_NS2	16.8	1.1	30.5	0.7	0.88	0.5	Neof./Rel. Olivine
WN-790_NS3	24.5	1.0	43.1	0.7	2.08	0.5	Neof. Olivine
WN-790_NS4	22.9	0.9	39.8	0.6	2.23	0.5	Neof. Olivine
WN-790_NS5	22.8	1.0	40.2	0.7	1.92	0.5	Neof. Olivine
WN-790_NS6	22.8	1.0	40.4	0.7	1.83	0.5	Neof. Olivine
WN-790_NS7	22.3	1.0	41.0	0.7	0.95	0.5	Neof. Olivine
WN-790_NS8	22.7	1.0	39.6	0.7	2.09	0.5	Neof. Olivine

Abbreviations: Rel. = relict; Neof. = neoformed; WN = Walnumfjellet nunatak; LK = Larkman Nunatak; GMM = Budel Collection

Table S2. **Chemical composition (element wt%) of Fe,Ni,S metal beads**

Analysis #	Si	Ni	S	Fe	Cr	Co	Total
DL	*0.00*	*0.01*	*0.01*	*0.02*	*0.01*	*0.02*	
WN-349_met	10.01	32.7	32.2	31.3	0.03	0.94	97.2
WN-349_met	20.14	32.1	31.6	30.6	0.04	0.95	95.4
WN-349_met	30.29	28.9	31.4	34.1	0.03	0.75	95.5
WN-349_met	40.21	33.4	29.2	27.5	0.03	1.29	91.7
WN-088_met1[a]	0.61	0.9	21.5	59.7	0.05	0.24	83.0
WN-790_met1[a]	1.23	16.9	18.1	39.2	0.03	0.66	76.1
WN-790_met2[a]	0.43	20.1	27.1	36.3	0.04	0.78	84.7

Abbreviations: DL = detection limit. [a] low total likely due to partial oxidation

Table S3. **Major and minor element composition (in oxide wt%) and fayalite (Fa) content of relict and neoformed olivine**. Data for particles WN-088, WN-349, WN-587, WN-464, WN-790 and LK06-0395.

Analysis #	SiO_2	Al_2O_3	Cr_2O_3	FeO	MnO	MgO	CaO	NiO	Total	Fa
DL	*0.05*	*0.03*	*0.05*	*0.06*	*0.06*	*0.02*	*0.02*	*0.06*		
Relict olivine										
**WN-088**										
WN-088_olR1	41.6	b.d.l.	0.25	1.6	0.26	54.4	0.05	b.d.l.	98.1	2
WN-088_olR2	41.8	0.08	0.28	3.5	0.33	52.7	0.09	b.d.l.	98.8	4
Avg.	*41.7*	*0.08*	*0.27*	*2.6*	*0.29*	*53.5*	*0.07*	-	*98.5*	*3*
S.D.	*0.2*	*0.00*	*0.01*	*1.0*	*0.03*	*0.8*	*0.02*	-	*0.3*	*1*
**WN-349**										
WN-349_olR1	38.9	0.09	0.37	20.7	0.16	41.3	0.20	b.d.l.	101.6	22
WN-349_olR2	40.2	0.04	0.31	13.6	0.21	46.8	0.14	b.d.l.	101.4	14
Avg.	*39.6*	*0.06*	*0.34*	*17.1*	*0.18*	*44.0*	*0.17*	-	*101.5*	*18*
S.D.	*0.9*	*0.02*	*0.03*	*3.5*	*0.02*	*2.8*	*0.03*	-	*0.1*	*4*
**WN-464**										
WN-464_olR1	39.8	0.11	0.31	14.5	0.19	45.1	0.21	0.09	100.2	15
WN-464_olR2	40.2	0.12	0.28	13.8	0.26	45.7	0.20	b.d.l.	100.6	15
WN-464_olR3	40.0	0.10	0.31	15.0	0.25	44.6	0.20	b.d.l.	100.4	16
WN-464_olR4	40.3	0.10	0.27	13.0	0.22	47.9	0.21	b.d.l.	101.9	13
WN-464_olR5	39.9	0.11	0.29	14.5	0.24	46.5	0.23	b.d.l.	101.8	15
WN-464_olR6	40.7	0.05	0.30	12.2	0.25	47.1	0.20	b.d.l.	100.7	13
WN-464_olR7	40.6	0.07	0.31	12.3	0.26	47.2	0.21	b.d.l.	101.0	13
Avg.	*40.2*	*0.09*	*0.30*	*13.6*	*0.24*	*46.3*	*0.21*	-	*101.0*	*13*
S.D.	*0.3*	*0.02*	*0.02*	*1.1*	*0.03*	*1.2*	*0.01*	-	*0.7*	*5*
**WN-587**										
WN-587_olR1	40.4	0.04	0.33	1.7	0.53	57.1	0.08	b.d.l.	100.2	2
**WN-790**										
WN-790_olR1	33.2	0.15	0.31	17.8	0.17	46.5	0.17	b.d.l.	98.4	18
WN-790_olR2	39.7	0.08	0.28	15.9	0.20	44.8	0.11	b.d.l.	101.0	17
WN-790_olR3	39.8	0.08	0.32	16.6	0.16	44.3	0.11	b.d.l.	101.4	17
WN-790_olR4	39.8	0.08	0.33	16.4	0.21	44.8	0.11	b.d.l.	101.7	17
WN-790_olR5	39.5	0.08	0.28	17.2	0.17	43.6	0.13	b.d.l.	101.0	18
Avg.	*38.4*	*0.09*	*0.30*	*16.8*	*0.18*	*44.8*	*0.13*	-	*100.7*	*17*
S.D.	*2.9*	*0.03*	*0.02*	*0.7*	*0.02*	*1.1*	*0.03*	-	*1.3*	*1*
**GMM-75**										
GMM75_olR1	39.3	0.01	0.12	15.8	0.33	45.3	0.16	b.d.l.	101.0	16

Table S3. Continued

**Analysis #**	**SiO_2**	**Al_2O_3**	**Cr_2O_3**	**FeO**	**MnO**	**MgO**	**CaO**	**NiO**	**Total**	**Fa**
**GMM-136**										
GMM136_olR1	41.1	0.02	0.72	7.0	0.57	51.5	0.14	b.d.l.	101.1	7
<u>Neoformed olivine</u>										
**LK06-0395**										
LK06-0395_olN1	38.1	0.09	0.36	20.9	0.20	39.8	0.31	0.20	100.0	23
LK06-0395_olN2	38.6	0.15	0.32	20.2	0.28	39.9	0.27	0.11	100.0	22
LK06-0395_olN3	37.0	0.21	0.42	28.6	0.41	33.6	0.38	0.30	101.0	32
LK06-0395_olN4	35.9	0.23	0.50	33.7	0.46	28.4	0.40	0.29	99.9	40
LK06-0395_olN5	37.2	0.13	0.29	28.8	0.40	33.8	0.35	0.28	101.3	32
LK06-0395_olN6	39.3	0.09	0.25	15.0	0.22	44.4	0.20	0.07	99.5	16
LK06-0395_olN7	38.8	0.12	0.35	20.4	0.24	39.3	0.30	0.08	99.7	23
LK06-0395_olN8	40.1	0.11	0.24	16.8	0.26	43.7	0.25	b.d.l.	101.5	18
LK06-0395_olN9	39.8	0.09	0.29	16.4	0.28	43.2	0.25	b.d.l.	100.2	18
LK06-0395_olN10	40.1	0.00	0.26	14.5	0.35	44.7	0.20	b.d.l.	100.1	15
LK06-0395_olN11	41.0	0.07	0.30	10.2	0.26	48.9	0.20	b.d.l.	100.9	10
LK06-0395_olN12	39.1	0.07	0.39	18.1	0.32	41.2	0.23	b.d.l.	99.3	20
LK06-0395_olN13	40.2	0.05	0.34	15.4	0.23	44.9	0.22	b.d.l.	101.2	16
LK06-0395_olN14	40.0	0.19	0.33	14.2	0.32	45.6	0.39	b.d.l.	101.0	15
LK06-0395_olN15	40.0	0.09	0.29	16.0	0.28	44.1	0.21	b.d.l.	101.0	17
LK06-0395_olN16	39.3	0.07	0.31	19.1	0.36	41.3	0.22	b.d.l.	100.6	21
LK06-0395_olN17	40.0	0.14	0.29	16.6	0.29	43.3	0.32	b.d.l.	101.0	18
LK06-0395_olN18	40.3	0.08	0.33	12.3	0.19	46.0	0.22	0.07	99.4	13
LK06-0395_olN19	38.8	0.03	0.36	20.1	0.47	39.7	0.22	b.d.l.	99.7	22
LK06-0395_olN20	40.8	0.03	0.27	11.0	0.29	47.3	0.17	b.d.l.	100.0	12
LK06-0395_olN21	40.4	0.07	0.34	13.0	0.34	46.2	0.17	b.d.l.	100.5	14
LK06-0395_olN22	38.5	0.30	0.22	21.9	0.37	39.3	0.47	b.d.l.	101.2	24
LK06-0395_olN23	38.9	0.08	0.38	18.7	0.24	41.1	0.21	b.d.l.	99.6	20
LK06-0395_olN24	39.6	0.10	0.32	16.5	0.30	43.8	0.23	b.d.l.	100.8	17
Avg.	*39.2*	*0.11*	*0.32*	*18.3*	*0.31*	*41.8*	*0.27*	*0.17*	*100.4*	*20*
S.D.	*1.2*	*0.07*	*0.06*	*5.7*	*0.08*	*4.7*	*0.08*	*0.10*	*0.7*	*7*
**WN-088**										
WN-088_olN1	38.1	0.10	0.59	20.2	0.48	38.5	0.12	0.07	98.1	23
WN-088_olN2	40.2	0.07	0.37	10.6	0.38	47.2	0.08	b.d.l.	98.9	11
WN-088_olN3	38.7	0.09	0.31	14.1	0.34	44.7	0.11	b.d.l.	98.3	15
WN-088_olN4	38.4	0.09	0.34	17.3	0.39	41.6	0.09	b.d.l.	98.2	19
WN-088_olN5	39.3	0.09	0.32	13.8	0.41	44.2	0.11	b.d.l.	98.2	15
WN-088_olN6	39.9	0.12	0.27	9.6	0.49	47.8	0.32	b.d.l.	98.4	10
WN-088_olN7	37.1	0.20	0.71	27.2	0.60	33.8	0.21	b.d.l.	99.8	31
WN-088_olN8	39.5	0.07	0.43	12.2	0.47	45.9	0.12	b.d.l.	98.7	13
WN-088_olN9	37.0	0.23	0.56	25.7	0.62	33.9	0.23	b.d.l.	98.3	30
WN-088_olN10	40.1	0.07	0.31	10.7	0.42	47.9	0.11	b.d.l.	99.6	11
WN-088_olN11	39.7	0.10	0.32	13.1	0.66	45.7	0.24	b.d.l.	99.9	14

Table S3. Continued

**Analysis #**	**SiO_2**	**Al_2O_3**	**Cr_2O_3**	**FeO**	**MnO**	**MgO**	**CaO**	**NiO**	**Total**	**Fa**
WN-088_olN12	39.6	0.08	0.39	14.6	0.49	45.2	0.13	b.d.l.	100.4	15
WN-088_olN13	39.4	0.19	0.34	13.1	0.50	45.9	0.11	b.d.l.	99.5	14
WN-088_olN14	29.8	1.71	1.28	54.8	0.81	11.5	0.37	0.09	100.4	73
WN-088_olN15	40.0	0.05	0.34	12.0	0.42	46.8	0.09	b.d.l.	99.7	13
WN-088_olN16	29.4	1.40	0.47	60.4	0.90	4.8	0.46	0.07	97.9	87
WN-088_olN17	38.5	0.08	0.41	17.0	0.43	42.3	0.10	b.d.l.	98.9	18
WN-088_olN18	38.1	0.14	0.45	22.0	0.53	38.7	0.15	b.d.l.	100.1	24
WN-088_olN19	39.6	0.26	0.37	16.3	0.43	42.0	0.27	b.d.l.	99.2	18
WN-088_olN20	34.5	0.21	0.59	37.3	0.59	25.0	0.16	b.d.l.	98.3	46
WN-088_olN21	38.0	0.34	0.29	16.4	0.55	43.0	0.16	b.d.l.	98.7	18
Avg.	*37.8*	*0.27*	*0.45*	*20.9*	*0.52*	*38.9*	*0.18*	*0.07*	*99.0*	*25*
S.D.	*3.1*	*0.44*	*0.22*	*13.9*	*0.14*	*11.7*	*0.10*	*0.01*	*0.8*	*20*
**WN-349**										
WN-349_olN1	37.8	0.11	0.34	22.0	0.24	38.5	0.20	b.d.l.	99.2	24
WN-349_olN2	39.3	0.06	0.23	15.2	0.30	43.9	0.29	b.d.l.	99.3	16
WN-349_olN3	38.0	0.13	0.32	21.4	0.26	39.8	0.22	b.d.l.	100.1	23
WN-349_olN4	38.9	0.16	0.30	14.5	0.24	44.4	0.28	b.d.l.	98.8	15
WN-349_olN5	38.5	0.07	0.36	19.6	0.28	40.8	0.19	b.d.l.	99.8	21
WN-349_olN6	31.7	0.25	0.54	56.1	0.54	9.0	0.69	b.d.l.	98.8	78
WN-349_olN7	33.9	0.30	0.50	49.2	0.52	15.9	0.37	b.d.l.	100.7	63
WN-349_olN8	38.7	0.08	0.29	14.5	0.19	44.4	0.13	b.d.l.	98.4	15
WN-349_olN9	38.8	0.09	0.44	17.5	0.22	42.9	0.18	b.d.l.	100.1	19
WN-349_olN10	37.7	0.11	0.33	21.6	0.22	38.5	0.18	0.09	98.7	24
WN-349_olN11	36.6	0.20	0.50	34.3	0.22	29.4	0.36	0.33	101.2	40
WN-349_olN12	39.8	0.08	0.33	16.9	0.30	44.7	0.30	0.12	102.5	18
WN-349_olN13	37.9	0.17	0.34	21.2	0.31	39.7	0.21	b.d.l.	99.8	23
WN-349_olN14	39.0	0.08	0.26	15.6	0.23	44.6	0.15	b.d.l.	100.0	16
WN-349_olN15	38.1	0.11	0.40	21.1	0.24	39.5	0.24	b.d.l.	99.6	23
WN-349_olN16	37.7	0.10	0.31	19.8	0.33	41.2	0.17	b.d.l.	99.6	21
WN-349_olN17	34.7	0.11	0.51	35.8	0.39	26.8	0.33	0.12	98.7	43
WN-349_olN18	39.4	0.09	0.32	13.2	0.23	46.6	0.13	b.d.l.	99.9	14
WN-349_olN19	38.8	0.09	0.34	15.1	0.32	43.6	0.24	b.d.l.	98.4	16
WN-349_olN20	34.0	0.26	0.42	43.2	0.33	21.8	0.45	0.27	100.8	53
WN-349_olN21	38.4	0.08	0.35	18.6	0.25	41.2	0.18	b.d.l.	99.1	20
WN-349_olN22	36.6	0.14	0.44	28.7	0.22	33.8	0.34	0.16	100.4	32
WN-349_olN23	31.0	0.30	0.27	56.9	0.59	8.3	0.82	b.d.l.	98.1	79
WN-349_olN24	34.0	0.21	0.64	40.1	0.50	24.2	0.35	0.11	100.1	48
WN-349_olN25	37.7	0.08	0.31	26.1	0.20	35.7	0.19	0.08	100.4	29
WN-349_olN26	37.8	0.08	0.37	25.8	0.20	36.5	0.22	0.11	101.2	28
WN-349_olN27	37.8	0.08	0.34	25.8	0.22	36.4	0.21	0.06	100.9	28
WN-349_olN28	37.9	0.08	0.36	26.7	0.22	35.6	0.23	0.06	101.1	30
WN-349_olN29	37.0	0.16	0.41	30.7	0.24	32.7	0.24	0.13	101.6	34
WN-349_olN30	34.9	0.30	0.58	41.6	0.26	23.4	0.41	0.33	101.8	50

Table S3. Continued

**Analysis #**	**SiO_2**	**Al_2O_3**	**Cr_2O_3**	**FeO**	**MnO**	**MgO**	**CaO**	**NiO**	**Total**	**Fa**
WN-349_olN31	34.2	0.34	0.56	45.7	0.33	19.4	0.52	0.43	101.5	57
WN-349_olN32	32.6	0.37	0.32	53.4	0.35	12.7	0.87	0.40	101.1	70
WN-349_olN33	35.1	0.27	0.36	41.8	0.26	23.2	0.47	0.26	101.7	50
WN-349_olN34	32.0	0.31	0.25	57.8	0.38	8.8	1.01	0.38	100.9	79
WN-349_olN35	39.4	0.04	0.29	13.0	0.24	46.4	0.25	0.07	99.7	14
Avg.	*36.7*	*0.16*	*0.38*	*29.1*	*0.30*	*33.0*	*0.33*	*0.19*	*100.1*	*35*
S.D.	*2.5*	*0.09*	*0.10*	*14.1*	*0.10*	*11.8*	*0.21*	*0.13*	*1.1*	*20*
**WN-464**										
WN-464_olN1	39.0	0.13	0.33	19.8	0.30	40.7	0.29	b.d.l.	100.6	21
WN-464_olN2	38.2	0.16	0.42	22.9	0.31	38.0	0.30	0.12	100.4	25
WN-464_olN3	37.9	0.16	0.40	22.6	0.31	37.8	0.31	b.d.l.	99.5	25
WN-464_olN4	37.1	0.19	0.39	25.0	0.31	34.8	0.31	0.06	98.2	29
WN-464_olN5	39.6	0.20	0.35	16.5	0.27	44.4	0.29	0.07	101.6	17
WN-464_olN6	39.2	0.30	0.37	19.7	0.23	41.6	0.47	b.d.l.	101.8	21
WN-464_olN7	39.0	0.32	0.33	20.9	0.30	41.0	0.50	b.d.l.	102.5	22
WN-464_olN8	38.7	0.21	0.39	22.1	0.27	39.9	0.41	0.07	102.1	24
WN-464_olN9	38.2	0.17	0.39	24.2	0.31	37.5	0.38	b.d.l.	101.1	27
WN-464_olN10	36.8	0.26	0.49	27.4	0.41	33.2	0.44	b.d.l.	99.0	32
WN-464_olN11	38.4	0.61	0.43	21.9	0.41	36.4	0.62	b.d.l.	98.7	25
Avg.	38.4	0.25	0.39	22.1	0.31	38.7	0.39	0.08	100.5	24
S.D.	0.9	0.14	0.05	2.9	0.05	3.2	0.11	0.02	1.5	4
**WN-587**										
WN-587_olN1	37.8	0.09	0.38	24.3	0.26	36.7	0.04	0.07	99.6	27
WN-587_olN2	36.6	0.12	0.42	29.8	0.24	31.4	0.07	0.13	98.8	35
WN-587_olN3	36.9	0.16	0.32	34.2	0.21	28.7	0.11	0.24	100.9	40
WN-587_olN4	35.2	0.26	0.26	41.4	0.22	22.4	0.18	0.42	100.4	51
WN-587_olN5	32.7	0.67	0.23	47.2	0.20	18.3	0.25	0.38	99.9	59
WN-587_olN6	39.6	0.16	0.30	16.2	0.24	43.9	0.07	b.d.l.	100.4	17
WN-587_olN7	28.9	1.34	0.08	61.5	0.26	7.0	0.54	0.52	100.0	83
Avg.	*35.4*	*0.40*	*0.28*	*36.4*	*0.23*	*26.9*	*0.18*	*0.29*	*100.0*	*45*
S.D.	*3.6*	*0.46*	*0.11*	*15.1*	*0.02*	*12.2*	*0.18*	*0.18*	*0.7*	*22*
**WN-790**										
WN-790_olN1	40.6	0.10	0.29	10.5	0.25	48.0	0.12	b.d.l.	99.8	11
WN-790_olN2	39.6	0.09	0.42	12.0	0.22	45.7	0.11	b.d.l.	98.2	13
WN-790_olN3	39.6	0.18	0.32	13.9	0.36	43.9	0.14	b.d.l.	98.4	15
WN-790_olN4	36.8	0.11	0.50	27.5	0.27	33.7	0.19	b.d.l.	99.0	31
WN-790_olN5	40.2	0.05	0.35	10.8	0.29	47.6	0.13	b.d.l.	99.4	11
WN-790_olN6	38.4	0.08	0.38	17.6	0.21	41.7	0.15	b.d.l.	98.6	19
WN-790_olN7	40.8	0.06	0.35	10.3	0.21	47.7	0.10	b.d.l.	99.5	11
WN-790_olN8	39.7	0.04	0.50	15.6	0.24	42.7	0.13	b.d.l.	98.9	17
WN-790_olN9	38.6	0.10	0.35	20.9	0.28	39.3	0.13	b.d.l.	99.6	23

Table S3. Continued

**Analysis #**	**SiO_2**	**Al_2O_3**	**Cr_2O_3**	**FeO**	**MnO**	**MgO**	**CaO**	**NiO**	**Total**	**Fa**
WN-790_olN10	35.9	0.12	0.43	33.2	0.37	29.0	0.26	0.09	99.4	39
WN-790_olN11	38.6	0.06	0.41	16.9	0.24	42.1	0.13	b.d.l.	98.4	18
WN-790_olN12	37.8	0.09	0.29	17.3	0.24	42.5	0.14	b.d.l.	98.4	19
WN-790_olN13	38.0	0.15	0.42	25.4	0.42	35.8	0.16	b.d.l.	100.3	28
WN-790_olN14	37.3	0.15	0.36	24.2	0.24	36.6	0.18	b.d.l.	99.0	27
WN-790_olN15	38.1	0.07	0.37	20.7	0.24	40.3	0.15	b.d.l.	100.0	22
WN-790_olN16	35.1	0.15	0.59	36.2	0.32	25.6	0.25	0.07	98.3	44
WN-790_olN17	36.8	0.09	0.38	26.6	0.25	35.6	0.20	b.d.l.	100.0	30
WN-790_olN18	36.1	0.13	0.40	33.3	0.44	30.6	0.25	0.15	101.4	38
WN-790_olN19	39.0	0.10	0.33	19.5	0.27	41.6	0.13	b.d.l.	100.9	21
WN-790_olN20	39.9	0.04	0.28	12.7	0.27	46.8	0.13	b.d.l.	100.1	13
WN-790_olN21	38.2	0.09	0.36	21.6	0.25	38.6	0.16	0.07	99.3	24
WN-790_olN22	36.2	0.13	0.38	31.0	0.26	31.6	0.19	b.d.l.	99.7	35
WN-790_olN23	33.0	0.95	0.46	40.8	0.30	22.8	0.51	b.d.l.	98.9	50
WN-790_olN24	35.4	0.16	0.40	37.2	0.32	25.6	0.23	b.d.l.	99.3	45
Avg.	*37.9*	*0.14*	*0.39*	*22.3*	*0.28*	*38.1*	*0.18*	*0.10*	*99.4*	*25*
S.D.	*1.9*	*0.18*	*0.07*	*9.2*	*0.06*	*7.5*	*0.08*	*0.04*	*0.8*	*12*
**GMM-59**										
GMM59_olN1	39.98	0.06	0.24	9.71	0.20	49.14	0.15	0.05	99.53	10
GMM59_olN2	38.90	0.10	0.34	18.55	0.25	41.26	0.07	0.05	99.55	20
Avg.	39.44	0.08	0.29	14.13	0.23	45.20	0.11	0.05	9.54	15
S.D.	0.76	0.03	0.07	6.25	0.04	5.57	0.06	0.00	0.01	7
**GMM-75**										
GMM75_olN1	41.75	0.83	0.41	11.07	0.33	46.01	0.48	0.01	101.2	12
GMM75_olN2	41.00	0.12	0.42	12.98	0.29	46.74	0.19	0.03	101.8	13
GMM75_olN3	40.72	0.09	0.34	11.27	0.25	47.61	0.10	0.02	100.5	12
Avg.	41.16	0.35	0.39	11.77	0.29	46.79	0.26	0.02	101.2	12
S.D.	0.53	0.42	0.04	1.05	0.04	0.80	0.20	0.01	0.65	1
**GMM-136**										
GMM136_olN1	37.7	0.09	0.43	23.3	0.25	39.5	0.12	0.06	101.5	25
GMM136_olN2	38.8	0.10	0.35	19.9	0.23	43.8	0.17	0.04	103.4	20
Avg.	38.2	0.10	0.39	21.6	0.24	41.6	0.15	0.05	102.4	23
S.D.	0.8	0.01	0.06	2.4	0.01	3.0	0.03	0.02	1.4	3

Abbreviation: DL = detection limit; Avg. = average; S.D. = standard deviation.

Table S4. **Major and minor element composition (in oxide wt%) of the glassy mesostasis**. Data for particles WN-088, WN-349, WN-790 and LK06-0395.

**Sample**	**Na_2O**	**Al_2O_3**	**K_2O**	**TiO_2**	**FeO**	**MgO**	**SiO_2**	**CaO**	**Cr_2O_3**	**MnO**	**S**	**P2O5**	**NiO**	**Total**
DL	*0.03*	*0.03*	*0.01*	*0.05*	*0.06*	*0.02*	*0.05*	*0.02*	*0.05*	*0.06*	*0.07*	*0.07*	*0.06*	
**WN-088**														
	b.d.l.	8.45	b.d.l.	0.39	29.3	5.56	47.0	5.13	0.70	1.14	0.74	0.19	b.d.l.	98.6
	b.d.l.	9.00	b.d.l.	0.42	28.0	5.17	48.0	5.21	0.83	1.07	0.41	0.12	b.d.l.	98.2
	0.04	9.62	0.02	0.48	33.2	2.89	44.6	5.95	0.61	0.99	1.10	0.15	b.d.l.	99.6
	b.d.l.	9.33	0.02	0.44	28.6	4.71	47.3	5.37	0.69	0.92	0.83	0.15	b.d.l.	98.3
	b.d.l.	9.93	b.d.l.	0.47	30.0	3.78	46.7	5.84	0.59	0.98	0.97	0.18	b.d.l.	99.5
	b.d.l.	10.79	b.d.l.	0.53	34.6	1.71	42.6	6.29	0.43	0.93	1.51	0.00	b.d.l.	99.4
Avg.	-	9.52	0.02	0.45	30.6	3.97	46.0	5.63	0.64	1.00	0.93	0.13	b.d.l.	98.9
S.D.	-	0.80	0.00	0.05	2.7	1.47	2.0	0.46	0.13	0.09	0.37	0.07	b.d.l.	0.6
**WN-349**														
	0.06	7.69	0.02	0.30	49.3	0.85	33.7	7.83	0.29	0.39	0.64	0.00	b.d.l.	101.0
	0.04	12.13	b.d.l.	0.49	37.5	0.35	34.9	12.09	0.06	0.30	1.52	0.00	b.d.l.	99.4
	b.d.l.	11.03	0.02	0.45	36.4	0.42	36.6	12.22	0.06	0.44	1.06	0.00	b.d.l.	98.6
	0.03	10.44	b.d.l.	0.39	28.1	4.17	47.8	9.41	0.41	0.41	0.53	0.00	b.d.l.	101.7
	b.d.l.	11.33	b.d.l.	0.47	44.5	0.51	30.1	10.95	0.08	0.30	1.84	0.00	0.10	100.2
Avg.	0.04	10.52	0.02	0.42	39.2	1.26	36.6	10.50	0.18	0.37	1.12	0.00	-	100.2
S.D.	0.02	1.70	0.00	0.08	8.1	1.64	6.7	1.87	0.16	0.07	0.56	0.00	-	1.2
**WN-790**														
	0.08	9.15	b.d.l.	0.72	33.4	2.87	43.6	6.53	2.54	0.58	0.71	0.16	b.d.l.	100.3
	0.03	9.32	b.d.l.	0.35	36.5	2.34	41.5	7.23	0.27	0.52	0.75	0.27	b.d.l.	99.1
	b.d.l.	9.87	b.d.l.	0.40	37.9	0.80	39.1	8.76	0.19	0.39	0.84	0.20	b.d.l.	98.4
	0.03	5.69	b.d.l.	0.22	47.9	6.02	35.5	4.48	0.31	0.47	0.15	0.26	b.d.l.	101.0
	b.d.l.	8.01	b.d.l.	0.37	41.4	1.30	37.9	8.35	0.52	0.31	0.24	0.25	b.d.l.	98.6
	0.05	7.93	0.03	0.32	22.6	7.14	52.8	6.26	0.75	0.54	0.15	0.16	b.d.l.	98.8
	b.d.l.	7.40	b.d.l.	0.32	24.4	8.55	50.4	5.37	0.76	0.54	0.14	0.12	b.d.l.	98.0
Avg.	0.05	8.20	-	0.38	34.9	4.14	43.0	6.71	0.76	0.48	0.42	0.20	-	99.2
S.D.	0.02	1.42	-	0.16	9.0	3.06	6.5	1.54	0.82	0.10	0.32	0.06	-	1.1

Table S4. Continued

Sample	Na_2O	Al_2O_3	K_2O	TiO_2	FeO	MgO	SiO_2	CaO	Cr_2O_3	MnO	S	P2O5	NiO	Total
**LK06-0395**														
	0.04	10.39	b.d.l.	0.31	36.2	1.06	38.9	12.97	0.09	0.41	0.27	0.00	0.12	100.8
	0.08	9.90	b.d.l.	0.44	38.2	0.41	37.6	13.67	0.13	0.42	1.79	0.00	b.d.l.	102.6
	b.d.l.	7.64	b.d.l.	0.27	42.4	2.59	37.8	8.60	0.30	0.59	1.26	0.00	b.d.l.	101.4
	0.06	8.96	b.d.l.	0.37	32.2	2.22	44.3	10.68	0.31	0.66	1.11	0.00	b.d.l.	100.9
	0.04	6.91	b.d.l.	0.33	30.0	5.02	47.1	9.22	0.77	0.64	0.64	0.00	b.d.l.	100.6
	0.04	7.94	0.02	0.31	28.6	4.46	48.5	9.41	0.49	0.57	0.44	0.00	0.07	100.9
	0.07	7.66	b.d.l.	0.32	27.7	4.30	48.2	9.70	0.65	0.59	0.31	0.00	b.d.l.	99.6
	b.d.l.	6.92	0.02	0.29	25.5	7.15	48.1	8.28	0.63	0.71	0.33	0.00	b.d.l.	98.0
	0.06	6.99	0.02	0.24	24.5	9.16	48.3	7.45	0.48	0.57	0.29	0.00	b.d.l.	98.1
	0.11	7.33	0.02	0.29	29.3	4.47	47.9	9.72	0.69	0.69	0.68	0.00	b.d.l.	101.2
	0.06	8.54	b.d.l.	0.36	32.9	1.77	43.1	11.49	0.31	0.46	1.10	0.00	b.d.l.	100.1
Avg.	0.06	8.11	0.02	0.32	31.6	3.87	44.5	10.11	0.44	0.57	0.75	0.00	0.10	100.4
S.D.	0.02	1.20	0.00	0.05	5.5	2.64	4.5	1.93	0.23	0.10	0.50	0.00	0.03	1.4
**GMM-59**														
	0.01	5.35	b.d.l.	0.22	39.22	9.06	40.76	2.45	0.83	0.57	0.65	0.07	0.01	99.18
	0.01	7.39	b.d.l.	0.40	36.87	4.62	43.47	3.55	0.75	0.60	1.07	0.10	0.01	98.81
Avg.	0.01	6.37	b.d.l.	0.31	38.05	6.84	42.11	3.00	0.79	0.59	0.86	0.09	0.01	99.00
S.D.	0.00	1.44	-	0.13	1.66	3.14	1.91	0.77	0.06	0.02	0.30	0.02	0.00	0.26
**GMM-75**														
	0.08	7.74	b.d.l.	0.41	32.05	5.67	45.76	4.68	1.44	0.73	1.80	0.27	0.00	100.64
	0.03	7.39	b.d.l.	0.38	30.68	6.13	47.19	4.83	0.94	0.71	1.25	0.23	-0.01	99.76
Avg.	0.06	7.56	-	0.40	31.36	5.90	46.48	4.76	1.19	0.72	1.53	0.25	-0.01	100.20
S.D.	0.04	0.25	-	0.02	0.97	0.32	1.01	0.11	0.36	0.02	0.39	0.03	0.00	0.62
**GMM-136**														
	0.00	2.88	b.d.l.	0.15	26.2	25.57	42.72	1.59	0.69	0.41	0.58	0.19	0.01	101.01

Abbreviations: DL = detection limit; Avg. = average; S.D. = standard deviation; b.d.l. = below detection limit.

Table S5. **EPMA defocused-beam major and minor element analyses (in oxide wt%) of ScumPo spherules.** Data for particles WN-088, WN-349, WN-790 and LK06-0395.

**Sample**	**Na_2O**	**Al_2O_3**	**K_2O**	**TiO_2**	**FeO**	**MgO**	**SiO_2**	**CaO**	**Cr_2O_3**	**MnO**	**S**	**P_2O_5**	**NiO**	**Cl**	**V_2O_3**	**CoO**	**Total**
DL		*0.03*	*0.02*	*0.01*	*0.04*	*0.05*	*0.02*	*0.04*	*0.02*	*0.04*	*0.05*	*0.03*	*0.06*	*0.06*	*0.01*	*0.06*	*0.05*
**WN-088**																	
	b.d.l	3.76	b.d.l	0.17	27.97	24.21	39.67	2.15	0.62	0.76	0.32	0.08	b.d.l	b.d.l	b.d.l	b.d.l	99.7
	b.d.l	2.07	0.02	0.17	38.01	25.63	31.23	0.90	0.37	0.47	0.45	0.07	0.08	0.06	b.d.l	0.10	99.6
	b.d.l	3.50	0.02	0.18	24.60	28.92	40.46	1.92	0.65	0.71	0.28	0.11	b.d.l	b.d.l	b.d.l	b.d.l	101.4
**WN-349**																	
	b.d.l	2.27	b.d.l	0.11	28.29	30.66	37.68	2.20	0.41	0.33	0.15	b.d.l	b.d.l	b.d.l	b.d.l	b.d.l	102.1
	b.d.l	1.66	0.02	0.07	36.76	28.66	33.61	1.56	0.42	0.25	0.54	b.d.l	0.14	0.01	b.d.l	b.d.l	103.7
	0.05	1.63	b.d.l	0.06	32.63	30.04	34.40	1.84	0.34	0.28	0.79	b.d.l	b.d.l	b.d.l	b.d.l	0.06	102.1
**WN-790**																	
	b.d.l	3.14	b.d.l	0.16	37.28	20.81	36.12	2.44	0.47	0.31	0.12	0.13	0.11	0.01	b.d.l	b.d.l	101.1
	b.d.l	2.07	b.d.l	0.07	33.27	27.86	35.96	1.54	0.44	0.34	0.20	0.10	b.d.l	0.02	b.d.l	0.11	102.0
**LK06-0395**																	
	b.d.l	5.59	b.d.l	0.25	28.60	14.05	44.44	6.85	0.67	0.55	0.45	b.d.l	b.d.l	b.d.l	b.d.l	b.d.l	101.4
	b.d.l	2.51	b.d.l	0.06	25.35	29.45	39.57	2.84	0.56	0.44	0.26	b.d.l	b.d.l	0.01	b.d.l	b.d.l	101.1
	b.d.l	1.91	b.d.l	b.d.l	27.79	31.60	38.31	2.40	0.40	0.38	0.16	b.d.l	b.d.l	b.d.l	b.d.l	b.d.l	102.9

Abbreviations: DL = detection limit; b.d.l. = below detection limit.

Table S6. **Compositions used for CY and CI Chondrites.**

	**Y-86720 (CY)**	**Ivuna (CI)**
SiO_2	39.3	33.6
TiO_2	0.1	0.0
Al_2O_3	3.0	2.9
FeO	19.9	18.9
NiO	2.1	1.5
Cr_2O_3	0.5	0.5
MnO	0.2	0.1
MgO	26.4	19.5
CaO	0.7	2.8
Na_2O	0.6	0.5
K_2O	0.1	0.2
P_2O_5	0.2	0.2
Total	93.0	90.2
